# The Dynamical Interplay of Collective Attention, Awareness and Epidemics Spreading in the Multiplex Social Networks During COVID-19

**DOI:** 10.1109/ACCESS.2020.3031014

**Published:** 2020-10-14

**Authors:** Marialisa Scatá, Barbara Attanasio, Grazia Veronica Aiosa, Aurelio La Corte

**Affiliations:** Dipartimento di Ingegneria Elettrica, Elettronica ed Informatica (DIEEI)Universitá di Catania 9298 95125 Catania Italy

**Keywords:** Multiplex networks, social networks, epidemics spreading, social contagion, collective attention, collective awareness, COVID-19

## Abstract

Leveraging social and communication technologies, we can digitally observe that the collective attention typically exhibits a heterogeneous structure. It shows that people’s interests are organized in clusters around different topics, but the rising of an extraordinary emergency event, as the coronavirus disease epidemics, channels the people’s attention into a more homogenized structure, shifting it as triggered by a non-random collective process. The connectedness of networked individuals, on multiple social levels, impacts on the attention, representing a tuning element of different behavioural outcomes, changing the awareness diffusion enough to produce effects on epidemics spreading. We propose a mathematical framework to model the interplay between the collective attention and the co-evolving processes of awareness diffusion, modelled as a social contagion phenomenon, and epidemic spreading on weighted multiplex networks. Our proposed modeling approach structures a systematically understanding as a social network marker of interdependent collective dynamics through the introduction of the multiplex dimension of both networked individuals and topics, quantifying the role of human-related factors, as homophily, network properties, and heterogeneity. We introduce a data-driven approach by integrating different types of data, digitally traced as user-generated data from Twitter and Google Trends, in response to an extraordinary emergency event as coronavirus disease. Our findings demonstrate how the proposed model allows us to quantify the reaction of the collective attention, proving that it can represent a social predictive marker of the awareness dynamics, unveiling the impact on epidemic spreading, for a timely crisis response planning. Simulations results shed light on the coherence between the data-driven approach and the proposed analytical model.

## I. Introduction

The outbreak of severe acute respiratory syndrome coronavirus 2 (SARS-CoV-2), a novel coronavirus, emerged in the city of Wuhan (Hubei, China) in early December of 2019, has posed a global public interest and it has raised concerns in most people worldwide on the future health and well-being [1]–[3]. This new betacoronavirus have shocked the world and has drawn global attention since it has caused a fast diffusion. The groups of cases of pneumonia of unknown cause detected in Wuhan was reported to the World Health Organization (WHO) on the 31st December 2019, and consequently this new virus was named 2019 novel coronavirus (2019-nCoV) [4] and SARS-CoV-2, by the International Committee on Taxonomy of Virus (ICTV) on the 11th February 2020, and the diseases caused by SARS-CoV-2 were named Corona Virus Diseases 2019 (COVID-19) on the same day [1], [4]. Since then, besides China, cases confirmed with COVID-19 had also been detected in many other countries and territories [4], [5], thus, from the city of Wuhan that has taken unprecedented measures in response to the outbreak, including extended school and workplace closures, also the other countries follows these strategies through various national responses to the pandemic, including measures such as lockdowns, quarantines, curfews and other restrictions (stay-at-home orders, shelter-in-place orders, shutdowns/lockdowns) aimed at preventing further spread of COVID-19. On 30th January 2020 WHO officially proclaimed the outbreak of COVID-19 as a public health emergency of international concern, and establishes temporary recommendations, early indications, giving a boost to a different reaction, especially in terms of timing. The public health measures for managing the possible outbreaks count on the preparedness ability of each country, namely the capacity to prevent, detect, verify, assess, and react in accordance with the International Health Regulations for COVID-19 [6], [7]. The majority of methods, which take into considerations evidence-based public health prevention strategies weighs the people’s interests and the ongoing development consciousness which makes it possible to shape the public awareness on behaviours [6]. The global public attention to this issue can reflect people’s interests and their propensity to acquire knowledge on COVID-19 with the aim at taking precautionary actions. In fact, to understand an emergency event, it is insufficient to rely on numerical reports based only on confirmed cases and the spatial spread [8], but it is needed adding more information, integrating knowledge and data on people’s behaviours to quantify other dominant variables that can influence and impact possible future outbreaks. Exploiting the social networks and communication technologies under this extraordinary emergency condition, a systematic understanding of social collective dynamics, based on the complex networks paradigms that integrate weighted multiplex networks mathematical representation, can be achieved. To do this, we need to detect how the public attention around COVID-19, could guide the situational awareness and the timing of intervention strategies, impacting on the epidemics spreading. A large corpus of scientific literature have studied the phenomenon of spreading deepen inside a better understanding on how it produces dynamics which depends on various structural and human-related factors [9], [10], suggesting that in social networks the dynamics depend on the nature of social ties [11]. Since behaviours [11], [12], misinformation [13], [14], infectious diseases [15], [16], distress [17], emotions [18] 0and competing processes [19], [20] spread through interactions of networked individuals, starting from classical epidemiological models [21]–[23], and based on an interdisciplinary approach which involves several research fields in complex network science [24]–[27], we are able to model the diffusion dynamics into the social networks. Taking into consideration the modeling approach of dynamics of social contagion and spreading processes in complex networks [16], [18], [28], we are interested to grasp the linkage among the shape of collective phenomena and their interpersonal spreading in networks structure, in response to a triggering force as an extraordinary event able to shift the common outcomes to unexpected peaks and clusters. Many studies have investigated the spreading phenomena and its co-evolution, as the awareness, modelled as social contagion phenomena and the epidemics spreading unveiling the interplay between them, in a multiplex network but separating and constraining each of the processes to only one of the layers [9], [29], [30]. By contrast, in [16], [17], it has been explored and quantified the impact of the co-evolution of the two processes in all the layers considering the introduction of the multiplexity dimension. Multiplex networks representation consider the same set of nodes in all the layers of interactions, and constitute the most suitable network structure to understand such dynamical processes and their complex interdependence [12], [31]–[33]. The interplay between epidemic spreading and awareness dynamics allows to highlight the role of structures and human-related factors in the multiplex networks which influence the dynamical trend of both processes. Since the more the networked individuals are aware about a disease spreading, the more they may be able to adopt strategies with consciousness, permitting the rising of collective awareness. In this paper, we propose an analytical and data-driven rigorous investigation of what is the shape of response in terms of collective attention around a shocking and long-lasting event in which awareness and attention, in single and collective terms, jointly develop in an interdependent way. We investigate and measure which is the role and the weight of multiple and heterogeneous social ties, through the introduction of the weighted multiplex network, and the impact on the epidemic spreading. We consider two weighted multiplex networks, in which we evaluate both the complex dynamics of nodes belonging to a population and that one referred to keywords, mined from social networks, and linked to the topic COVID-19. As social ties between nodes may have different weights reflecting their intensity [34], we consider the two weighted multiplex networks providing new definitions of weights for both multiplexs. We include, in the weights of population-based multiplex, the homophily and the awareness difference between nodes, which in turn depends on the collective attention dynamics of the keywords-based multiplex, that considers the keywords co-adoption interactions, defined in our model. We quantify the impact of the attention and awareness on epidemic spreading, by introducing heterogeneity, both in terms of susceptibility and awareness, for optimally schedule effective crisis communications, facilitating timely crisis response planning, such as the decision of a time warning and quarantine. It is crucial to examine how the attention and the awareness arise and fade in different communities, affected in various times, impacting and influencing behaviours and decisions. Moreover, we define a data-driven approach applied in the case of COVID-19, referred to social networks and social media platforms user-generated data, as Twitter and Google Trends [1], [35]. We compare our findings with a null model, to investigate a time-dependant and complex relationship of attention and awareness centred on the ongoing epidemics of COVID-19.

### A. Contributions of This Paper

Once explained the motivations behind the proposed methodology, the main contributions of the paper are summarised below.

• We propose a modeling and data-driven approach to structure a systematic understanding of collective attention dynamics of social networks, during an extraordinary event for a better crisis response. We point out to unveil the dynamical interplay between collective attention and two co-evolving spreading processes of awareness and epidemics on weighted multiplex networks.
• We disclose the role of human-related factors, such as homophily, namely the tendency of interacts with similar users, network properties and heterogeneity through the introduction of two interdependent weighted multiplex networks which respectively include connections of networked individuals, based on theoretical and empirical interactions, and connections between keywords, based on the co-adoption relationships. The empirical interactions between users and the co-adoption interactions between keywords are mined from the user-generated data digitally traced from Twitter and Google Trends, during the COVID-19 epidemics.
• We define weights between users of the first weighted multiplex network by taking into account both the concept of homophily and awareness, pointing out how these weights influence the epidemics dynamics on the social multiplex network. For the second weighted multiplex network, we define the interactions weights based on the co-adoption of a pair of keywords, as the frequency of their combined use by users digitally traced through the data-driven approach.
• We explore and quantify the dynamical interplay of collective attention on COVID-19 with the co-evolving spreading processes, defining the awareness measures of the users, as a measure depending on their participation coefficient, and the entropy of the topics, traced and analyzed in a specific temporal window and mined from social and communications technologies.
• We quantify the reaction of collective attention, detecting a social network predictive marker of the awareness dynamics in social networks and its key role on a timely crisis response planning and management of the epidemics spreading of COVID-19.

## II. Related Works

### A. Epidemics Spreading and Awareness as Social Contagion in Multiplex Networks

The complex systems are fully described by their connectedness and the network representation of the nodes, belonging to them, which interact to each other via multiple links. We can describe a network through a standard approach, consisting of the analysis of the aggregate graph, that includes all links between nodes but neglecting important information, resulting in a losing knowledge about the structural complexity and connectivity. In fact, the relationships and interactions between nodes in many real-world systems can be different for relevance, context and meaning and can be characterized by a distance or weight [31], [32], [34]. To preserve the knowledge related to the different interactions in multiple layers we need to introduce the multiplex dimension of the network. It enables us to better quantify information encoded in terms of collective and social behaviours and in terms of spreading and diffusion. Multiplex networks, in which nodes can be adjacent to each other, through intra-layer edges or to its counterpart on another layer through the inter-layer ones [31], [32], represent the most suitable network structure for analyzing the emerging dynamical patterns of spreading phenomena, depending on the nature of social ties [17], [36]. The investigation of a multi-dimensional network representation through the multiplex networks enable us to fully characterize the behaviour of a complex system, unveiling interesting structural properties that helps to understand emerging phenomena such as cascading failures, super-diffusion, spreading and epidemic dynamics [17], [37]–[42]. These networks allow us to encompass multiple interactions exploring in what measure, the ties in the different layers, have an impact on the diffusion of social phenomena. The importance of this mathematical representation is such that the scientific interest around multiplex networks applications ranges between a multitude of different areas: biological, social and technological systems, social networks and relationships, epidemic and social contagions, air transportation networks and brain computing dynamics [11], [16], [17], [37]–[39], [41]. Complexity, in connections, can be explored in a deeper way in structural terms taking into considerations weighted multiplex networks [34], since in real-world context, the social ties between nodes may have different weights reflecting their intensity in the different layers. For that assumption, the links between nodes not only are distinguished by the kind of interaction linking the nodes, but also by the intensity reflecting the importance of these interactions. Since we are interested in capturing the impact that the dynamics of collective attention around a real event has on the co-evolution of epidemic spreading and awareness,and differently from the above cited scientific literature, we firstly investigate the role of weighted multiplex networks by defining specific weights of the ties. In addition, with regards to the complexity in emerging dynamics of the co-evolving and interdependent spreading processes, in the weighted multiplex networks, we include the analysis of social contagion. Social contagion is modelled as a spreading process of diseases [16], and it is based on epidemiological models [21], and it finds applications in network science, social behavioural analysis, misinformation diffusion, infectious disease and emotions spreading [11]. These phenomena spread inter-personally, and, thanks to a vast amount of literature that has investigated these complex dynamics, following classical epidemiological models [21]–[23], involving several research fields in network science [24]–[27], [43]–[46], we can state that the nature of social ties has a key role in a phenomenon diffusion on a social network [11], [14], [18], [28], [47], [48]. Several studies investigate the role of the awareness in the disease spreading process, disclosing his effect on the networked individuals more likely to adopt strategies targeted at self-protecting [16], [17] to slow diffusion, if they are more aware about it. Many studies have explored the spreading and competition of both phenomena separating them into different layers of diffusion [9], [30], [49]–[51]. Otherwise, since the multiplex networks constitute the most fit network structure to study these processes, their interdependence and co-evolution [33], we follow the approach to do not separate each of the spreading process to only one layers, but as in [16], [17] we investigate and quantify the impact of the co-evolution of the two processes in all the layers of the weighted multiplex network, adding the collective attention influence on the awareness dynamics fitness during an extraordinary event as COVID-19 epidemics. Coherently with the real nature of multiplex networks [36], it has been taken into account heterogeneity and its impact along with awareness on the epidemic spreading [16], [52].

### B. Collective Attention

A growing number of existing studies have investigated the concept of collective attention, especially when it comes from the user-generated data. Since the escalation of social and communication technologies provides everyday a chance to digitally observe the interests of networked individuals, there is a growing involvement by the researchers in leveraging these technologies with the aim at providing predictors useful in case of crisis response [35]. Social media, and major communication platforms is having a significant impact on broadening participation by social networked individual, facilitating the contribution of information, ideas, contents, opinions, that we can see as a marker of their interests [35], [53]. The heterogeneity in terms of information loaded in the communications platforms, in conjunction with the heterogeneity in terms of network structure, impact on the user attention, showing that if the collective attention is limited, this can leads to a low discriminating capacity for users in understanding the quality of information [53]. Other studies have investigated how the collective attention in the online social platforms can represent a digital fingerprint of the human behaviours. It is interesting to underline that, when a real-world event occurs, the activity, in terms of participation or content sharing, which represents the collective attention on social media, exhibits a burst-like increase and an irregular oscillation, otherwise it follows a regular circadian rhythms, as showed in [54]. Moreover, as investigated in [35], with a comparison between originally observed attention graphs and a baseline, it is notable that collective attention displays a heterogeneous structure, showing that the interests of networked individuals generally are polarized around clusters, with reference to different topics. When an emergency or shocking event occurs, there is a shifting on people’s attention inducing a convergence into a more homogeneous structure. The events attract the attention of people impacting on the awareness of them, changing the social behaviour and, in some cases, it depends more from the content exposure and much less by the event dynamics, such as in case of an epidemics [55]. Although the concept of collective attention remains not well defined and it is related to different quantities and parameters, such as burstiness or popularity in content sharing [51], [56], [57], in several works, authors decided to focus on the emergence of collective attention through the analysis of contents streams from Twitter communications, to detect the shape of the dynamics [35], [53], [54]. Other works are based on Google Trends, which enables to analyze the popularity of specific search terms [58], [59]. Recently, around the emergency linked to the COVID-19, in [1], such as in case of other health emergencies [60], the authors studies the overall search trends regarding the epidemics, which increase in the early time of the observation period, varying in each country considered. To systematically understand the digital traces of the collective attention released and the interplay with the individual awareness which in turns produces changes in behaviours, [11], [30], [45], we need to discover and quantify the dynamics of the interdependence, using both theoretical frameworks and empirical data. To this aim, we propose an analytical framework based on the heterogeneous weighted multiplex networks structure and the data-driven approach which allow us to integrate and collect data from Twitter communications and Google Trends. In this way we are able to detect a possible social network marker to forecast the trend of awareness dynamics and its impact on the epidemics, exploiting the infodemics of online social platforms and the connectedness of users.

## III. Model

### A. Scenario

**FIGURE 1.**
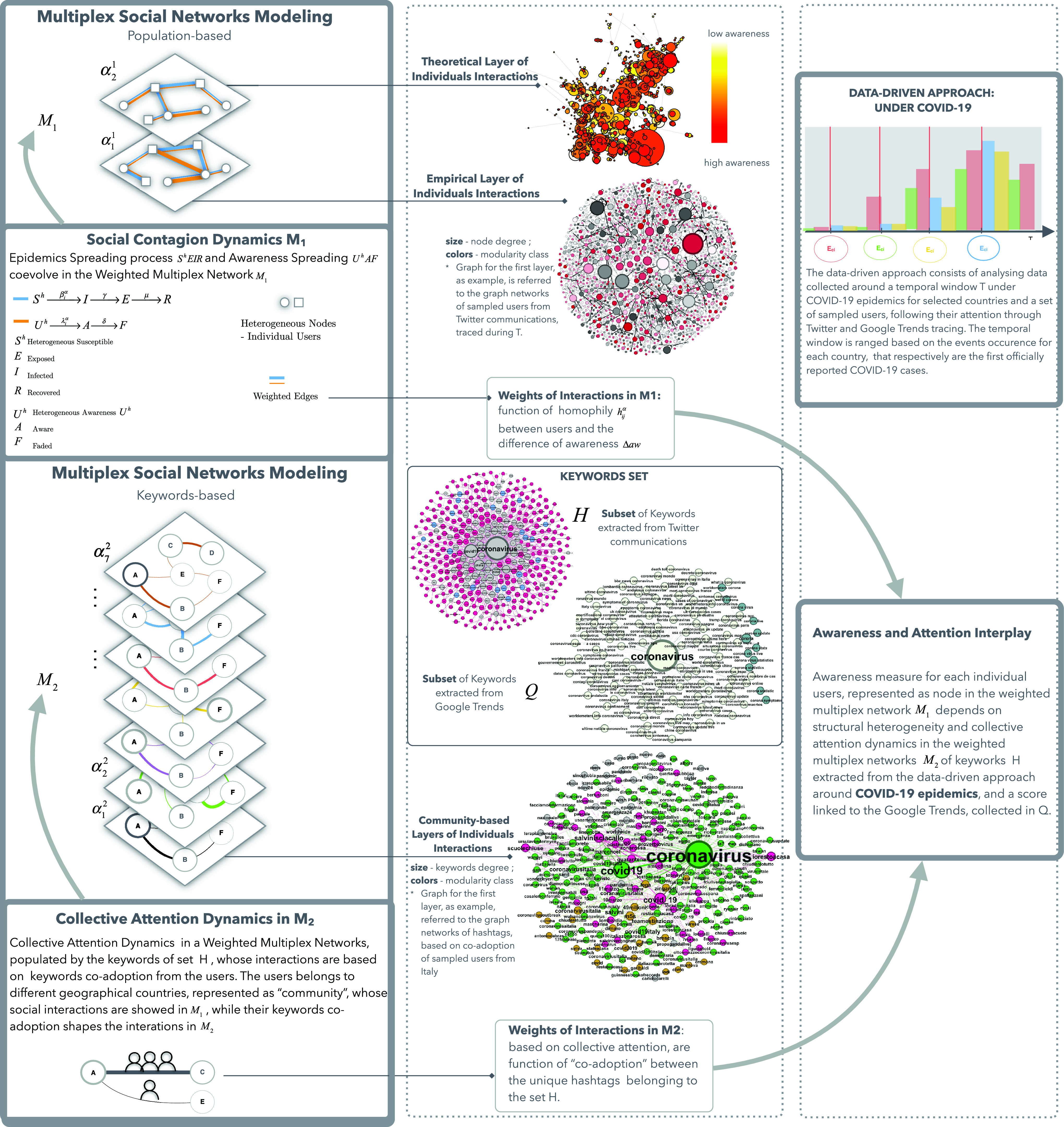
From Modeling to Data-driven approach. The figure schematically describes the various steps and key aspects starting from the modeling procedure to the data-driven approach. In the left side of the framework we highlight the modeling steps, in which we define two weighted multiplex social networks, population-based and keywords-based, with their dynamics, respectively, a social contagion dynamics of two co-evolving spreading processes, epidemics and awareness, and the collective attention. In the right panel we show the data-driven approach, specifically around the COVID-19 epidemics. The outcomes of the latter approach and analysis are showed in the central panel, in which some of the entries are empirically extracted from the Twitter communications and from Google Trends. Moreover, we underline the interplay and the interdependence between awareness and attention which link in a complex co-evolution the dynamics of the two weighted multiplex social networks, allowing us to disclose the joint impact of attention and awareness on epidemics, and helping us to forecast how the individuals react to the emergency.

We introduce a new framework, as showed in Fig. 1, to quantify the interplay between collective attention and awareness dynamics, modeling them in two co-evolving and interdependent weighted multiplex social networks. Centering the analysis around the COVID-19, we choose geographical countries, represented as “communities”, which have encountered the first case of COVID-19, within a fixed time window $T$. The $T$ interval has been properly selected to extract the relevant spreading dynamics in different countries. Each first episode of COVID-19, officially reported in each selected geographical countries, here is stated as “event occurrence” $E_{c_{i}}$ in $T$, with $c_{i}$, the country belonging to the set $C$ of countries selected. Following the data-driven and sampling approaches, presented in the Sections IV-A, the users, belonging to the communities, constitute the nodes which populate the first multiplex social network $M_{1}$, namely the population-based multiplex. Each layer of $M_{1}$ is referred to different kinds of interactions based on social networks relationships. For this reason, in $M_{1}$, the interactions in the first layer between users, follow the theoretical scheme of a scale-free network [10], [61], with a power-law dependence of the degree distribution $P(k)-k^{\gamma }$, with the exponent $\gamma =2$ that typically satisfying values around $2 < \gamma < 3$. Instead, the second layer of $M_{1}$ follows the graph network extracted from the data-driven approach, showing virtual mined relationships for the same set of users. As detailed in the Section IV-A, we integrate and analyze large social networks communication by using Twitter datasets and Google Trends. We construct a set of keywords $K$, mining a set $H$ of unique hashtags from Twitter, related to the sampled relevant users (see IV-A3), and the set $Q$ of the most popular terms of searches from Google Trends, in the fixed temporal window $T$. The elements of the subset $H$ of $K$, represent the nodes of the population of the second weighted multiplex networks $M_{2}$,namely the keywords-based multiplex network. In $M_{2}$, each layer is a community-based level, and it is referred to a kind of interaction, that we define as “co-adoption” relationships, based on the combined use of keywords by any users of that community representing the layer, whose social interactions are explored in the multiplex network $M_{1}$. Following, for each weighted multiplex social networks, we detail the mathematical representation, the definitions of weights, by highlighting statistical estimators and the metrics, which impact on the co-evolution of collective awareness and attention, and the impact on the epidemic spreading.

### B. Multiplex Social Networks Modeling

#### 1) Population-Based

With regards to the first weighted multiplex social network, we highlight the importance of including multiple relationships between users, representing people of different communities as explained in the section III-A. The first multiplex network $M_{1}$, allowing to investigate how different network structure impact on the contagion dynamics, consists of two layers of interactions of the same set of users, a scale free-networks and a graph extracted from the data-driven approach. This makes us able to show a theoretical and an empirical interactions scheme to represent and investigate two types of virtual relationships for the same set of users. Let us consider the first weighted multiplex social network of $M_{1}$ layers $\alpha _{1} = \{ 1,\ldots, M_{1}\}$ and $N_{1}$ nodes $i=\{ 1,\ldots, N_{1}\}$. The multiplex network is a set of $M_{1}$ weighted networks (or layers) defined as a set of graphs $G$, vertices $V$ and edges $E$, ${G_\alpha } = (V,{E_\alpha })$ (see Fig 1). The set of nodes $N_{1}$ is the same for each layer, whereas the set of links $E_{1}$ changes according to the layer [34]. Each network ${G_\alpha }$ is described by the adjacency matrix, denoted by ${A^{\alpha }}$ with elements ${a^{\alpha }_{ij}}$, where ${a^{\alpha }_{ij}} = w_{ij}^\alpha > 0$, if there is a link between $i$ and $j$ with a weight $w_{ij}$, otherwise ${a^{\alpha }_{ij}} = 0$. We include a definition of weights as function of the discrepancy of users awareness about the referred topic ($\Delta aw$) of COVID-19 and homophily ($h_{ij}^\alpha $), namely the tendency of interacts with similar users.

Definition 1 (Weights of Interactions in *$M_{1}$*): Referred to the first multiplex network, the weights denoted as $w_{ij}^{\alpha }$ are given by:

\begin{equation*} w_{ij}^\alpha {|_{M_{1}}} = h_{ij}^\alpha \cdot \left |{ {\Delta aw} }\right | +1\tag{1}\end{equation*}

where $h_{ij}={1}/{1+\delta _{ij}}$ is the homophily between nodes $i$ and $j$. Homophily is defined as the tendency to associate and interact more with similar people [36], where $\delta _{ij}^\alpha $ is the measure of the homophily difference between nodes $i$ and $j$ as expressed in [36] and $\Delta aw$ is equal to the absolute difference of awareness, $\left |{{aw_{i} - {aw_{j}}}}\right |$, between nodes $i$ and $j$ (see III-B3). Homophily represent the principle that similarity breeds connections, enabling the structural understanding of how social connections are forged over time, [36]. It stands for an overall value which takes into account different dimension of similarity. Following the representation of that different dimension as different homophily values, we choose to indicate with $\delta _{ij}$ the homophily difference between a pair of nodes and with $\sigma $ its standard deviation.

#### 2) Keywords-Based

With reference to the second multiplex network, we underline the role of “keywords” as broker for users’ involved topics. We characterize the second weighted multiplex social networks $M_{2}$, populating it with keywords as nodes, and the multiple interactions, different for each layer, between nodes as the “co-adoption” relationships. We consider each layer of this multiplex network referred to each geographical country and the coexistence of several types of interactions among keywords based on the collective attention of individuals mined from social networks. Let us consider the second multiplex network of $M_{2}$ layers $\alpha _{2} = \{ 1,\ldots, M_{2}\}$ and $N_{2}$ nodes $i=\{ 1,\ldots, N_{2}\}$. The set of nodes is the subset $H$ of $h$ hashtags, linked to the main topic under investigation. As explained below, in section IV-A, we construct that set, mining a set of unique hashtags from Twitter, used by any users in the fixed temporal window $T$. In $T$, based on a data-driven approach, we analyze a large corpus of datasets and we digitally investigate and measure the collective attention. As underlined for $M_{1}$, we can also describe $M_{2}$ by the adjacency matrix, denoted by ${A^{\alpha }}$ with elements ${a^{\alpha }_{ij}}$, where ${a^{\alpha }_{ij}} = w_{ij}^\alpha > 0$, if there is a link between $i$ and $j$ with a weight $w_{ij}$, otherwise ${a^{\alpha }_{ij}} = 0$.

Definition 2 (Weights of Interactions in *$M_{2}$*): Regarding to the second multiplex network, the weights are denoted as $w_{ij|M_{2}}^{\alpha }$. To mathematically define the interaction weights, since they are based on the “co-adoption” relationships, as explained before, we introduce the hashtags “co-adoption” probabilities as relative frequencies, that are: $p^{\alpha }_{h_{i}h{j}}$ which denotes the relative frequency of using both hashtags by users belonging to the community of the layer $\alpha $ of $M_{2}$, and $p^{\alpha }_{h_{i}}$ as the relative frequency of using the hashtags $h^{\alpha }_{i}$, by users belonging to the same community. In accordance to the latter, the weights of interactions in $M_{2}$ are given by:

\begin{equation*} w_{h_{i}{h_{j}}|{M_{2}}}^\alpha = {p_{h_{i}{h_{j}}}} \cdot ({p_{h_{i}}} - {p_{h_{j}}})\tag{2}\end{equation*}

where, $h_{s}$ with $s$ in $\{1,2,\ldots H\}$ are hashtags elements belonging to a subset of hashtags $H$, linked to COVID-19, the main topic under investigation.

#### 3) Awareness and Attention Interplay

The awareness represents the acquired knowledge as a result of reasoning and understanding and, eventually, experiencing the epidemics. To capture the linkage between awareness and attention dynamics, which in turns impacts on the epidemics patterns, in this paper, we assume that the “awareness” measure, $aw_{i}$, for each node $i$ belonging to users population of weighted multiplex network $M_{1}$, depends on structural heterogeneity, collective attention dynamics on $M_{2}$, and node properties [31], [34], [62]. Moreover, we shed light on that awareness measure has not change according to the layers, since awareness once acquired is the same in the different layers in which a user is involved. It can be influenced by the fading of interests on acquiring additional and correlated awareness, or verifying if it is fact-checked or misinformation based [17], [63]. The awareness gap between two interacting nodes in $M_{1}$, impacts on the weights of links as detailed in section III-B1, in conjunction with the homophily value $h_{ij}$. In fact it is crucial to include the tendency to interact with similar people, through the introduction of homophily measure since strongly homogeneous groups tend to prefer contents that confirm their shared beliefs, polarizing rumours or misinformation. This phenomenon is defined as “echo chambers” effect which have a strict interplay with the spread of misinformation [63]. In presence of misinformation and highly homophilic clusters of users in social networks, it is very likely having fake news masqueraded as fact-checked contents [64].

Definition 3 (Awareness Measure in *$M_{1}$*): Referred to the first multiplex network $M_{1}$, the awareness measure for each node $i$, is denoted as $aw_{i}$, and it is given by:

\begin{equation*} a{w_{i_{t=T}}} = \left({{P_{i}}|_{M_{1}} \cdot \sum \limits _{h \in H}{{H_{h}}}\cdot \sum \limits _{q \in Q}{\eta _{q}}}\right) +a{w_{i_{t=t_{0}}}}\tag{3}\end{equation*}

where $P_{i}$ is the multiplex participation coefficient which enable us to include the heterogeneity of the number of neighbours of node $i$ across the layers in $M_{1}$ [31]. The $H_{h}$ represents the Shannon entropy of the hashtags $h$, mined from Twitter communications, belonging to the set $H$, which represents the population of nodes in $M_{2}$ [31], [34], and $\eta _{q}$ is a score, represented by the relative search volume (RSV), associated with the Google search popularity of terms $q$, elements of the subset $Q$ extracted through Google Trends (see section IV-A).

### C. Social Contagion Dynamics

#### 1) Discussing the Applicability of SEIR Model in Co-Evolution With the UAF Model for COVID-19

We study the spreading of two processes in $M_{1}$ in co-evolution in the same multiplex network, referred to the epidemic and the awareness spreading [16]. Deciding to not disjoint the two processes we are able to explore the emerging complex dynamics in order to highlight the effect of the awareness dynamics, which is marked by cognitive limitations, on epidemic spreading, underlining also its impact on how we are likely to accept the unprecedented restrictive measures due to the COVID-19. In fact, in response to the epidemic outbreak [2], [65]–[67] both local and national governments have taken non-pharmaceutical physical distancing interventions, strategies to reduce its impact, extended school closures and workplace distancing along with emergency spreading in time [68]. This complex dynamics investigation can shed light on the role of awareness in this emergency event, unveiling its interdependence with collective attention, keeping a watchful eyes on the fact-checked news, that can distance the natural memory leaks or fading of interest by human brain on epidemics. It is nevertheless worth mentioning that COVID-19, as it is clearly visible observing the timeline of pointed key occurred events [5], differently from shocking peaked events, like they could be earthquakes or mass shooting [35], it still observed it is an unfolded emergency in time. This raises the challenge of quantifying the impact of awareness, influenced by the dynamics of people interests, in terms of searching for information and content adoption, that is revealed by the analysis of the growing number of users-generated data [35], [54], [56], [69]. To this aim, we explore how dynamics could change according to network structure heterogeneity and awareness, which in turns depends on the attention dynamics explored in $M_{2}$. To detect a marker of the complex social dynamics, we adopt a data-driven approach which exploits large social media platform communications and searches as Twitter [35], [56] and Google Trends [59]. A rising number of studies explored the model to fit mathematically the transmission of COVID-19, taking into consideration measures and strategies which reduces social mixing, modifying both the pattern within the population and the trajectory of the epidemics. Since the transmission is mostly driven by who interacts with whom, which can vary by age, location, contact, the protective measures of distancing have been designed to shift the social mixing patterns [2]. Given that, most of these limitations transform population mixing as well as human habits and the real social connectedness, consequently it becomes crucial the understanding to what extent the attention and the awareness in social networks can lead to behaviours in accordance with the provided reduction strategies. For that reason, currently, the increasing virtual connectivity [1], [64], [70], expressed in multiple layers, can marker the realization of a collective consciousness and impacting on the COVID-19 epidemic progression. In accordance with the recent epidemiological studies on parameters of the outbreak and propagation [71], [72], [72]–[75], we consider the susceptible-exposed-infected-recovered (SEIR) model [2], [21], [72], as the epidemiological model for COVID-19, analyzing it in conjunction and co-evolution with the awareness social contagion process $UAF$ [16], in $M_{1}$. The SEIR model is generally used to model influence-like-illness, and it has been used to numerically analyze the evolution of the Severe Acute Respiratory Syndrome (SARS) in different social settings [21]. As observed in [71], the SEIR model fits mathematically with the nature of COVID-19 transmission. This model is a variation of the SIR model, with the inclusion of exposed state ($E$) [21]. Moreover, in line with [2], [21], [73], we assume that, Exposed state ($E$) is a state in which individuals have been infected by the disease but cannot yet transmit it. In [72], the authors consider a SEIR model with different assumptions, since they indicate as $E$ the latent state in which the population is asymptomatic but infectious, and $I$ referred to the symptomatic and infectious population. The incubation rate $\sigma $ is described as the rate by which the $E$ individual develops symptoms. In [74], instead, the authors named the proposed model as SIDARTHE discriminating between detected and undetected cases of infection and between different severity of illness (SOI), non-life-threatening cases (asymptomatic and pauci-symptomatic; minor and moderate infection) and potentially life-threatening cases (major and extreme). In our work, we have assumed that asymptomatic cases represent a minor proportion of infectiousness compared with symptomatic cases, following the hypothesis in accordance with [2], [73]. In fact, although the contributions of asymptomatic or sub-clinical cases represent a pivotal point, and the question of whether such individuals are able to transmit infection remains unresolved at the time of writing, we do not consider that cases in this work, in accordance with the choice of temporal window that was aim at tracing the initial phase of rising the interest of collective attention around COVID-19. To analyze the co-evolving dynamics of both epidemics and awareness spreading on $M_{1}$ multiplex network, we exploit the Dynamic Microscopic Markov Chain Approach (MMCA) (see section III-C3). Following our assumptions, we do not consider all demographic changes, age, structure features and other relevant compartments in population [21].

#### 2) Co-Evolving Spreading Processes

In accordance with what we have defined in the previous section, here we start from the key assumption that each node in $M_{1}$ has a different awareness on COVID-19 as underlined in section III-B3. As a consequence, each node, will be heterogeneously susceptible to the epidemics spreading, since the awareness acquired has an influence on the behaviours of people, improving their knowledge about epidemics. Heterogeneity and awareness are introduced in order to describe the spreading scenario and extricate the complex co-evolution of the interdependence, from one hand, between epidemics and awareness and, from the other hand, between awareness and attention (see section III-B3). We analyze two co-evolving spreading processes in the first weighted multiplex network $M_{1}$ (see section III-B1). The first process of epidemic spreading is indicated by $S^{h}EIR$, where $S^{h}$ indicates the heterogeneous susceptible state, $E$ the exposed state and $I$ and $R$, respectively the infection and the recovered ones [16], [21]. The second is the awareness spreading process, coexisting and co-evolving with the first one, indicated as $U^{h}AF$, that stands for “Unaware-Aware-Faded” model. The awareness spreading process is thought as a SIR-like, where $U$ indicates the condition of heterogeneous unawareness state, $A$ is the aware state, while the $F$ state, is the faded state in which the nodes, tend to decrease the interest to the referred topic, over time up to the point that it completely vanishes. The Awareness state is referred to the case of those who is aware on topic, which means that a user acquires knowledge about epidemics, as a result of a collective attention dynamics in social networks. This can leads to a discriminating capacity in understanding quality of good information on epidemics. In our model, the awareness state is not directly correlated to the ensuring that users follow quarantine or other physical strategies. Although an increasing of awareness can infers a good behaviour in line with that strategical measures,influencing people to follow preventive measures as distancing interventions [16], [17], [71], in our work it has been intended as a knowledge and a consciousness that can contrast for example the misinformation diffusion about COVID-19. The node, in the $F$ state, maintain the same awareness measure, but it has no interest in increasing its acquired knowledge on the phenomenon linked to the referred topic. Starting from the model presented in [16], we extend it introducing, other than the heterogeneity in terms of nodes’ susceptibility and awareness, in the layers of $M_{1}$ multiplex network, also the linkage to the expressed collective attention which feeds and drives the shape of awareness measure. The meaning of the various states of the two spreading processes are explained in Table 1, and a complete list of symbols with its fair meaning is summarized in Table 2, as follows:

**TABLE 1:** MMCA - States. We Include the Definitions of the Different States in the MMCA, Adding More Details About the Interdependence Between Epidemics, Awareness and Attention (See section III-C)

Status	Description
$S^{h}$	Heterogeneous Susceptible - Those who can contract the infection with a different infection rate. The nodes’ heterogeneity, influenced by the awareness rate, impacts on the probability to have a transition in exposed status.
$E$	Exposed - Those who have been infected but cannot yet transmit it.
$I$	Infected - Those who contracted the infection and are contagious.
$R$	Recovered - Those who recovered from the disease.
$U^{h}$	Heterogeneous Unaware - Those who is unaware of the epidemic, or with minimum or wrong awareness. The probability to have a transition in Aware state is different for each node, depending on the impact of the collective attention, whom dynamics is analyzed in $M_{2}$, on the weights of interactions of the multiplex network $M_{1}$.
$A$	Aware - Those who is aware of epidemic, or who develop an awareness measure based on fact-checked information. The awareness measure depends on the collective attention on epidemics (see section III-B3)
$F$	Faded - Those who tend to decrease the attention on epidemics, or the interest to improve knowledge. The more susceptible are nodes that reach the faded state, the more vulnerable they become.

**TABLE 2:** List of Symbols

Symbol	Description
$T$	Observed Time Interval.
$t_{i}$	Time sub-intervals considered.
$DB_{t}$	Datasets collected.
$C$	Set of locations $c_{i}$ considered
$E_{c_{i}}$	Event occurrence with $c_{i}$ the country belonging to $C$.
$H$	Set of unique hashtags $h$ from Twitter.
$Q$	Set of the most popular terms of searches $q$ from Google Trends.
$K$	Set of keywords $K = H \cup Q$.
$U$	Set of all users $u$.
$N$	Set of Sampled users.
${A^{\alpha }}$	Adjacency Matrix with elements ${a^{\alpha }_{ij}}$.
$M_{1}$	Population-based Multiplex Network.
$M_{2}$	Keywords-based Multiplex Network.
$aw_{i}$	Awareness of a node $i$ in the Multiplex Network $M_{1}$
$w_{i,j}^\alpha {|_{M_{1}}}$	Weights of interactions between two nodes $i$ and $j$ in a layer $\alpha $ of the Multiplex Network $M_{1}$
$w_{h_{i},h_{j}}^\alpha {|_{M_{2}}}$	Weights of interactions between two hashtags $h_{i}$ and $h_{j}$ in a layer $\alpha $ of the Multiplex Network $M_{2}$
$h_{i,j}$	Homophily between node $i$ and node $j$.
$p_{h_{i}}$	Relative frequency to use the hashtag $h_{i}$.
$p_{h_{i},h_{j}}$	Relative frequency to use both hashtags $h_{i},h_{j}$.
$P_{i}$	Multiplex Participation Coefficient of node $i$ in $M_{1}$.
$H_{h}$	Shannon entropy of hashtag $h$ in $M_{2}$.
$\eta _{q}$	Score associated with the RSV from GTrends.
$Y_{i}^\alpha $	Inverse Participation Ratio in the layer $\alpha $.
$s_{i}^\alpha $	Strength of node $i$ in the layer $\alpha $.
$o_{i}$	Overlapping degree of a node $i$.
$Z_{o_{i}}$	Z-score of a node $i$.
$\beta _{i}^{\alpha }$	Heterogeneous Infection rate.
$\lambda _{i}^{\alpha }$	Heterogeneous Awareness rate.
$\delta $	Fading rate.
$\gamma $	Probability to transit from state $E$ to state $I$
$\mu $	Probability to transit from state $I$ to state $R$
$q_{i}(t)$	Probability not being infected at step $t$.
$r_{i}(t)$	Probability not being aware at step $t$
$\beta _{c}$	Contagion Threshold.
$\rho $	Density of Infected nodes.

##### a: Heterogeneous SEIR

The ${S^{h}}EIR$ spreading process is diagrammatically expressed, in terms of reaction-diffusion equations, as follows:

\begin{equation*} {S^{h}}EIR \Rightarrow {S^{h}}\overset {\beta _{i}^{\alpha }}{\rightarrow } E \overset {\gamma }{\rightarrow }I\overset {\mu }{\rightarrow }R\tag{4}\end{equation*}

with $\beta _{i}^{\alpha }$, the infection rate for each node $i$ at each layer $\alpha $ in the multiplex network $M_{1}$. The probability of an exposed individual becoming infectious is $\gamma $, while the probability of becoming recovered after infection is equals to $\mu $. The heterogeneous infection rate represents the probability of a susceptible-infected contact results in a new exposure, as following expressed:

\begin{equation*} \beta _{i}^\alpha = \frac {1}{1 + \lambda _{i}^\alpha } \cdot \frac {1}{Y_{i}^\alpha }\tag{5}\end{equation*}

It depends on the network structure heterogeneity, through the measures of inverse participation ratio $Y_{i}^\alpha $ [34] (see Supplementary Information) and the rate of awareness $\lambda _{i}^{\alpha }$ as expressed in Eq. 7.

##### b: Heterogeneous UAF

The second spreading process is diagrammatically expressed as follows:

\begin{equation*} {U^{h}}AF \Rightarrow U\overset {\lambda _{i}}{\rightarrow }A\overset {\delta }{\rightarrow }F\tag{6}\end{equation*}

with $\lambda _{i}^{\alpha }$ and $\delta $, defined as transition rates, respectively represent the rate of awareness for each node $i$ at each layer $\alpha $ of the multiplex $M_{1}$ and the fading rate. The rate of awareness is expressed as follows:

\begin{equation*} \lambda _{i}^\alpha = \frac {s_{i}^\alpha }{1+s_{i}^\alpha }\cdot \lambda\tag{7}\end{equation*}

with $s_{i}^\alpha $ the strength of node $i$ in the layer $\alpha $ of the multiplex networks $M_{1}$ [34] (see Supplementary Information). The Dynamic Microscopic Markov Chain Approach (MMCA), enables us to explore the spreading dynamics of co-evolution on the weighted multiplex network $M_{1}$, depending also from the collective attention dynamics in $M_{2}$. In the next subsection we give a more detailed explanation of the MMCA used in our model.

#### 3) Dynamic Microscopic Markov Chain

**FIGURE 2.**
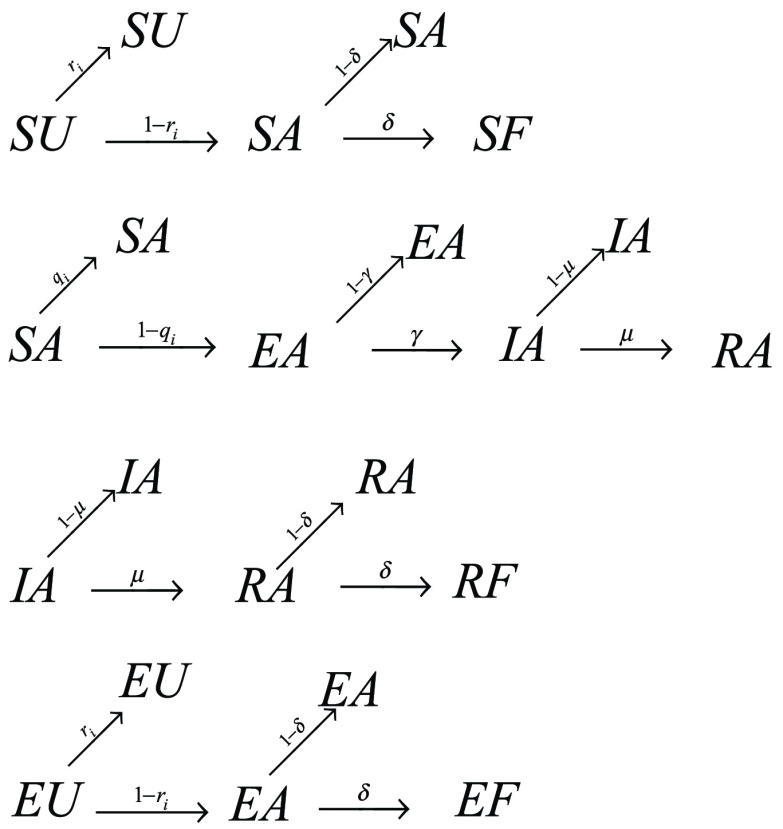
Probability tree. We show the probability tree, linked to the MMCA method, representing the states and the transitions, at each time step. Roots represent the initial states, SA, SU, IA and EA at time step t, while leaves are all the possible states at the subsequent time step. Arrows are labelled with the corresponding transition probabilities.

We start to assign to each node a state probability to be in one of the initial state, since at the beginning it can occupy only one of the following: susceptible and unaware (SU), susceptible and aware (SA), infected and aware (IA), exposed and unaware (EU) (see Fig. 2). Some states are not reachable or do not exist, such as infected and unaware (IU) and recovered and unaware (RU), due to the assumption that if a node has been infected or recovered has experienced the disease, developing a measure of awareness (see Fig. 2). At time step $t$ each node $i$ can occupy one of the initial states, with probabilities $p_{i}^{SU}(t)$, $p_{i}^{SA}(t)$, $p_{i}^{IA}(t)$, and $p_{i}^{EU}(t)$ respectively. We consider, as in [17], $q_{i}(t)$ as the probability of node $i$ not being infected at time step $t$ and $r_{i}(t)$ the probability of an unaware node $i$ staying unaware at time step $t$, as follows:

\begin{align*} {q_{i}}(t)=&(1 - {\overline \beta _{i}})\prod \limits _{j} {[1 - {a_{ji}}p_{j}^{I}(t){\overline \beta _{i}}]} \tag{8}\\ {r_{i}}(t)=&(1 - {\overline \lambda _{i}})\prod \limits _{j} {[1 - {a_{ji}}p_{j}^{A}(t){\overline \lambda _{j}}]}\tag{9}\end{align*}

where $a_{ij}$ are the elements of the adjacency matrix of each layer of the weighted multiplex network $M_{1}$. ${\overline \beta }_{i}$ and ${\overline \lambda _{i}}$ respectively are the mean values of the heterogeneous infection and awareness rate. The following MMCA equations represent the probability of each node of being in one of the states at time step $t+1$, as showed in Fig. 2:

\begin{align*} p_{i}^{SA}(t + 1)=&{q_{i}}(t)p_{i}^{SA}(t) + (1 - {r_{i}}(t))(1 - \delta)p_{i}^{SU}(t); \\ p_{i}^{SU}(t + 1)=&{r_{i}}(t)p_{i}^{SU}(t); \\ p_{i}^{SF}(t + 1)=&\delta (1 - {r_{i}}(t))p_{i}^{SU}(t); \\ p_{i}^{IA}(t + 1)=&\gamma (1 - {q_{i}}(t))(1 - \mu)p_{i}^{SA}(t) \\&+\,(1 - \mu)p_{i}^{IA}(t)); \\ p_{i}^{EU}(t + 1)=&{r_{i}}(t)p_{i}^{EU}(t); \\ p_{i}^{EA}(t + 1)=&(1-\delta)(1-{r_{i}}(t))p_{i}^{EU}(t) \\&+\,(1-\gamma)(1-{q_{i}}(t))p_{i}^{SA}(t); \\ p_{i}^{EF}(t + 1)=&\delta (1 - {r_{i}}(t))p_{i}^{EU}(t); \\ p_{i}^{RA}(t + 1)=&\mu (1 - \delta)p_{i}^{IA}(t) \\&+\,\gamma \mu (1 - {q_{i}}(t))(1-\delta) p_{i}^{SA}(t); \\ p_{i}^{RF}(t + 1)=&\mu \delta p_{i}^{IA}(t)+\delta \gamma \mu (1 - {q_{i}}(t))p_{i}^{SA}(t);\tag{10}\end{align*}

To obtain the contagion threshold, we investigate the steady state solution of the system constituted by the previous equations. When time $t \rightarrow +\infty $, there exists a contagion threshold $\beta _{C}$ for the two co-evolving processes, so that the contagion can outbreak only if $\beta \geq \beta _{C}$. Following the mathematical approach presented in [16], the contagion threshold is given by the order parameter $\rho _{i}$ and it is defined as follows:

\begin{equation*} {\rho ^{I}} = \frac {1}{N}\sum \limits _{i = 1}^{N} {p_{i}^{I}} = \frac {1}{N}\sum \limits _{i = 1}^{N} {p_{i}^{IA}}\tag{11}\end{equation*}

Thus, starting from equation $p_{i}^{IA}(t + 1)$ (see Eq. (10)), at steady state we have:

\begin{equation*} p_{i}^{IA} = \gamma (1-\mu)(1 - {q_{i}})p_{i}^{SA}\tag{12}\end{equation*}

Since around the contagion threshold $\beta _{C}$, the informed probability is close to zero ($p_{i}^{IA} = {\eta _{i}} \ll 1$), the probabilities of being informed can be approximated as follows:

\begin{equation*} {q_{i}} = (1 - \overline {\beta _{i}})\left[{1 - \overline {\beta _{j}} \sum \limits _{j} {{a_{ij}}\eta _{j}} }\right] = (1 - \overline {\beta _{i}})(1 - {\omega _{i}})\tag{13}\end{equation*}

where:

\begin{equation*} {\omega _{i}} = \overline {\beta _{j}} \sum \limits _{j} {{a_{ij}}{\eta _{j}}}\tag{14}\end{equation*}

Furthermore, close to the contagion onset we have that the fading rate is approximately close to zero ($\delta \simeq 0$). Considering this approximation into Eq. 12 and omitting higher order items, Eq. 12 is reduced to the following form:

\begin{equation*} \mu {\eta _{i}} \simeq \gamma (1-\mu)p_{i}^{SA}\overline {\beta _{i}} \overline {\beta _{j}} \sum \limits _{j} {{a_{ij}}{\eta _{j}}}\tag{15}\end{equation*}

The contagion threshold is obtained starting from the following condition:

\begin{equation*} \sum \limits _{j} {\left |{ {\gamma (1-\mu)\overline {\beta _{i}} p_{i}^{SA}{a_{ij}} - \frac {\mu }{{\overline {\beta _{j}} }}{t_{ji}}} }\right |{\eta _{j}} = 0}\tag{16}\end{equation*}

where $t_{ji}$ are the elements of the Identity matrix. By defining the matrix $H$ whose elements are given by: ${h_{ij}} = [\gamma (1-\mu)\overline {\beta _{i}} p_{i}^{SA}]{a_{ij}}$, the contagion threshold $\beta _{c}$ is the value corresponding to the largest eigenvalue of the matrix $H$, which is given by ${\Lambda _{\max }}(H) = \gamma \mu / \overline {\beta _{j}}$, so finally we get: ${\beta _{c}} = \gamma \mu / {\Lambda _{\max }(H)}$. The onset of the epidemic is the minimum value satisfying the Eq. 16. By denoting with ${\Lambda _{\max }(H)}$ the largest eigenvalue of $H$, we obtain the critical point of $\beta _{c}$ which depends explicitly on the co-evolving dynamics. Even if we consider the critical value referred to the onset of the awareness spreading dynamics $\lambda _{c}$ as a simple spreading process, when decoupled from the epidemic, the $(\beta _{c},\lambda _{c})$ defines a sort of meta-critical point for the spreading dynamics. In our work, the threshold model depends on the complex dynamical interplay, since the values of $\beta $ change also accordingly with the awareness rate, the network structure of the weighted multiplex network, the collective attention and the double heterogeneity in terms of susceptibility and awareness.

## IV. Experiment

### A. Data-Driven Approach

#### 1) Characterizing Collective Attention and Awareness Interplay, Under COVID-19

In our model, we propose a data-driven approach for evaluating the complex dynamics of co-evolving epidemics and awareness spreading, in function of the collective attention. To this aim, in the weighted multiplex network $M_{2}$, as showed in section III-B2, we analyze user-generated data and searches, respectively, by using a large corpus of Twitter communications datasets as listed in Table 3, and the most popular Google search terms, under COVID-19. The vast communications streams and searches, which still going on, enables us to monitor collective attention and, through the proposed framework in this paper, understand how it manifests itself under a real-world emergency event. In the whole temporal window considered $T$, we include small ranges $\Delta {t_{E_{c_{i}}}}$ created as a consequence of relevant events happened, referred to the time in which the event has been officially reported in the $c_{i}$ geographical countries considered, as detailed in section III-A. In particular, we take into account seven short sub-intervals, as listed in Table 4, in order to monitor attention patterns around the first officially reported cases of COVID-19 in each geographical country considered. Due to the observation constraints of Twitter API, in order to evaluate a large corpus of data in a long time window we exploited data collected in [76], [77], whose statistics are shown in Table 3. These datasets were created searching for users who have applied hashtags related to COVID-19 such as: #coronavirus, #coronavirusoutbreak, #coronavirusPandemic, #covid19, #covid_19 [76], [77]. Each tweet in the datasets includes textual content, the author id and nickname, the creation time, if it was in reply to another tweet, whether it is a retweet and additional metadata. To identify all topic, we extracted from tweets’ text all hashtags adopted and we considered both original tweets and retweets. In order to have a representation of the social network where the hashtags’ diffusion takes place and to estimate the activity of users around COVID-19 emergency, we traced back users, through the Twitter REST API, and we collected additional information such as locations and number of Followers (see Supplementary Information for further details). As showed in section III-A, we take into consideration two interdependent weighted multiplex networks to disclose the interplay between epidemics and awareness spreading, based on collective attention dynamics. We mined the “Retweet-Mention-Reply” graph of a group of relevant users, that through the sampling approach, becomes the sampled set $N$ of unique users, population of the weighted multiplex network $M_{1}$.We also construct the subset of the most relevant hashtags $H$ which represent the population of the weighted multiplex network $M_{2}$, restricting our data to the unique hashtags used by users in $N$ in all states under observation (see Supplementary Information). For these two weighted multiplex networks, we analyze the collective dynamics and interactions of the population of sampled users $N$ for $M_{1}$, and hashtags belonging to the subset $H$ for $M_{2}$. In order to examine the attention dynamics, we evaluate also the set $Q$ mined from Google Trends considering the top 25 search keywords, having the relative search volume (RSV) score greater than 0, and in particular, among these, the related queries of searches about “COVID-19” in each geographical country for each time interval $t_{i}$ in $T$. The pseudo code of the sampling and modeling approach and the social network marker detection is shown in Algorithm 1.

**TABLE 3:** Datasets Used in This Study

Dataset	Period	Users	Tweets Volume	Hashtag Volume
[76]	01-Dec-2019 −28-Feb-2020	41,914	60,160	10,986
[77]	01-Mar-2020 −11-Mar-2020	22,1928	526,791	135,664
[77]	12-Mar-2020	223,187	497,299	110,999
[77]	13-Mar-2020	569,099	994,611	238,367
[77]	14-Mar-2020	146,960	465,506	145,612
[77]	15-Mar-2020	245,218	464,396	142,924
[77]	16-Mar-2020	251,134	612,481	190,967
[77]	17-Mar-2020	356,262	834,944	249,083
[77]	18-Mar-2020	220,564	626,206	209,202
[77]	19-Mar-2020	284,981	855,845	267,057
[77]	20-Mar-2020	403,761	758,621	253,082
[77]	21-Mar-2020	393,600	881,955	256,816
[77]	22-Mar-2020	284,692	700,932	216,159
[77]	23-Mar-2020	255,576	658,225	229,724
[77]	24-Mar-2020	297,203	620,860	226,176
[77]	25-Mar-2020	278,293	684,556	245,815
[77]	26-Mar-2020	195,235	881,278	209,368
[77]	27-Mar-2020	302,559	664,120	239,108
[77]	28-Mar-2020	271,370	582,055	211,808
[77]	29-Mar-2020	227,820	564,141	204,568
[77]	30-Mar-2020	285,320	586,262	224,554

**TABLE 4:** Time Windows

t1	12-Dec-2019 - 31-Dec-2019
t2	01-Jan-2020 - 15-Jan-2020
t3	16-Jan-2020 - 31-Jan-2020
t4	01-Feb-2020 - 15-Feb-2020
t5	16-Feb-2020 - 29-Feb-2020
t6	01-Mar-2020 - 15-Mar-2020
t7	16-Mar-2020 - 30-Mar-2020

Algorithm 1 **The Interplay of Collective Dynamics in Multiplex Social Networks**
Input: Event in $T$ (as COVID-19); Datasets collected ($DB_{t}$); $\delta _{ij}$, $\mu $, $\delta $,$\gamma $.
Output: $M_{1}$,$M_{2}$, $aw_{i}$,$\beta _{c}$, $\rho _{I}$ in $T$.
1: **Phase 1: Sampling Approach.**
Sampled Set of Users $N \xleftarrow {} \emptyset $
Sampled Set of Hashtag $H \xleftarrow {} \emptyset $
Sampled Set of Queries $Q \xleftarrow {} \emptyset $
2: Set $t_{i} \in T$ filtering $DB_{t}$ by Tweets’ creation time.
3: $\forall t_{i} \in T$, $\forall u \in U$ collect data about users through Twitter API.
4: $\forall t_{i} \in T$, $\forall c$ collect queries through GTrends API.
5: $\forall t_{i} \in T$ and $\forall u \in U$ calculate “activeness” and “connectedness” of users, considering the number of tweets $n_{T_{u}}$ of a user $u$ and a threshold $T_{T}$, the number of followers $n_{F_{u}}$ of a user $u$ and a threshold $T_{F}$.
**if** $\forall t_{i} \in T$, $n_{T_{u}} > T_{T}$ **AND** $n_{F_{u}} > T_{F}$ **then** $N = N \cup u$.
6: $\forall t_{i} \in T$ and $\forall u \in N$ retrieve Hashtags from Tweets in $DB_{t}$.
7: Filter $N$ by location, $c$, and consider $H_{c}$ set of retrieved Hashtags.
8: Create $H = H_{1} \cap H_{2} \ldots \cap H_{c}$, and $Q = Q_{1} \cap Q_{2} \ldots \cap Q_{c}$ from the most frequent queries $\forall t_{i} \in T$.
9: Mining of the “Retweet-Mention-Reply” graph of the users in $N$ and the $c$ “Hashtags co-adoption” graphs of the hashtags in $H$.
10: Calculate the relative frequency of the hashtags in $H$, $p_{h}$.
11: **Phase 2: Multiplexity - Social Contagion.**
12: Set $N$ as $N_{1}$ population of $M_{1}$.
13: Set the “Retweet-Mention-Reply” graph of the users in $N$ as $G_{\alpha =1}$ and a Scale-free networks as $G_{\alpha =2}$, with $G_{\alpha }$ graphs of the $M_{1}$ with $\alpha ={1,\ldots,M_{1}}$ layers.
14: Set $H$ as $N_{2}$ population of $M_{2}$ multiplex network and the “Hashtags co-adoption” graphs of the users in $N$ as $G_{\alpha =c}$, with $G_{\alpha }$ graphs of the $M_{2}$ with $\alpha ={1,\ldots,M_{2}}$ layers.
15: $\forall i \in N_{1}$ in $M_{1}$ calculate $k_{i}$, $o_{i}$, $P_{i}$, $Z_{o_{i}}$.
16: $\forall h \in H$, in $M_{2}$, calculate calculate $k_{h}$, $o_{h}$, $H_{h}$ and $\forall q \in Q$ a score $\eta $ for each $c$ location.
17: $\forall i \in N_{1}$,in $M_{1}$, calculate $aw_{i}$, $w_{ij}$, $s_{i}^\alpha $, $Y_{i}^\alpha $
18: Calculate $w_{h_{i}h{j}}$ in $M_{2}$.
19: $\forall i \in N_{1}$, in $M_{1}$, assign to $i$ one of the initial states SU - SA - IA.
20: at time step $t$, calculate $\lambda ^\alpha _{i}$, $\beta ^\alpha _{i}$, $q_{i}(t)$, $r_{i}(t)$.
21: MMCA method.
22: Calculate $\beta _{C}$,$\rho _{I}$ in $T$.
23: **Phase 3: Social Network Marker.**
24: detect the emergence of the first event case $E_{c}^{I}$, and $\forall c$, detect $E_{c}$,$R_{t}^{c}$, $SM_{t}^{c}$, and calculate the delays $D_{R_{t}^{c}E_{c}^{I}}$, $D_{cm_{t}^{s}E_{c}}$ and $D_{R_{t}^{c}E_{c}}$.
25: $\forall c$, calculate $aw_{r}$, and, its growth rate $\pm aw_{r}(\%)$ as the social network marker impact.

#### 2) Sampling Approach

To construct the networks structure of the $M_{1}$ weighted multiplex network (see section III-B1), we consider a population of users, interacting through a scale-free network in the first layer and a sampled weighted graph network in the second layer, mined from a selection of a set of interacting nodes on Twitter and based on the datasets analyzed [76], [77]. Although a straightforward way for an understanding of attention dynamics is to gather data from as many users as possible, we assume that it is fundamental to apply a sampling approach, to identify the most influential users and their relationships, preserving the topological properties of the original structure. This set of sampled users, are that more likely can trigger awareness diffusion giving an impact on epidemic spreading. An ideal sample set should consider users with the following features [35]:

• *Activeness*: We examine in the sampled set, users who tend to tweet with hashtags linked to COVID-19, at a relatively high frequency during the time of interest $T$. In this way we include who have the tendency to maintain a high level of interest on topic through time, presuming that they are more likely to be active in the spreading dynamics. A user is included in the sampled set $N$ if has posted a number of tweets greater than a threshold $T_{T}$ in each time window $t_{i}$.
• *Connectedness*: We examine and include in sampled set, users who tend to be actively connected with other users showing their capability to cover a broader set of users, namely those who actively join into the common interests of many other users. We define the threshold $T_{F}$ of users’ number of followers and we add to the set N of sampled users who has a “followers_count” value greater than $T_{F}$.

Evaluating both the activeness and the connectedness we select the most popular users that are at the same time, the most active within the time period considered, avoiding missing content or activity gaps over time.

#### 3) Comparison With a Null Model

An interesting pivotal point is bringing together the nature of the extraordinary event, such as the one represented by COVID-19, with the usual emerging dynamical patterns from social networks. Without any doubt, this epidemic is not a common health emergency. In terms of collective attention, the interest is spread over time and continues to involve the population as a result of strategical measures and an uninterrupted updates on the data and the evolution of spreading itself [68], [70]. To evaluate if the emerging dynamical patterns is caused by an interplay between polarized attention and awareness on epidemics, or by a change on individual interests dynamics, rippling with a similar shape in other cases, we compare the observed dynamics with that one based on a null model. The null model is created for a fixed set of users, comparable to those considered in our model, in a period of time, prior to COVID-19, and comparable with our time interval $T$, during which we analyze the same set of data required to match the dynamical patterns. In this way, we can observe how and in what measure the observed dynamics differs from a baseline.

### B. Simulation Results and Discussion

Simulations have been carried out considering two weighted multiplex networks, respectively $M_{1}$ and $M_{2}$ as explained in Sections III-B1 and III-B2. Firstly, with regards to the $M_{1}$ weighted multiplex social network, we consider the key role of multiple relationships between users, which represent people of different communities, referred to the seven states considered in the data-driven approach. In $M_{1}$, we take into consideration the population of $N=1461$ users, representing the users of the sample extracted as showed in Section IV-A. In the first layer the interactions are based on the graph network extracted from the data-driven approach, showing virtual relationships for the sampled set of users, while in the second one the interactions follow the theoretical scheme of a scale-free network [10], [61].

**FIGURE 3.**
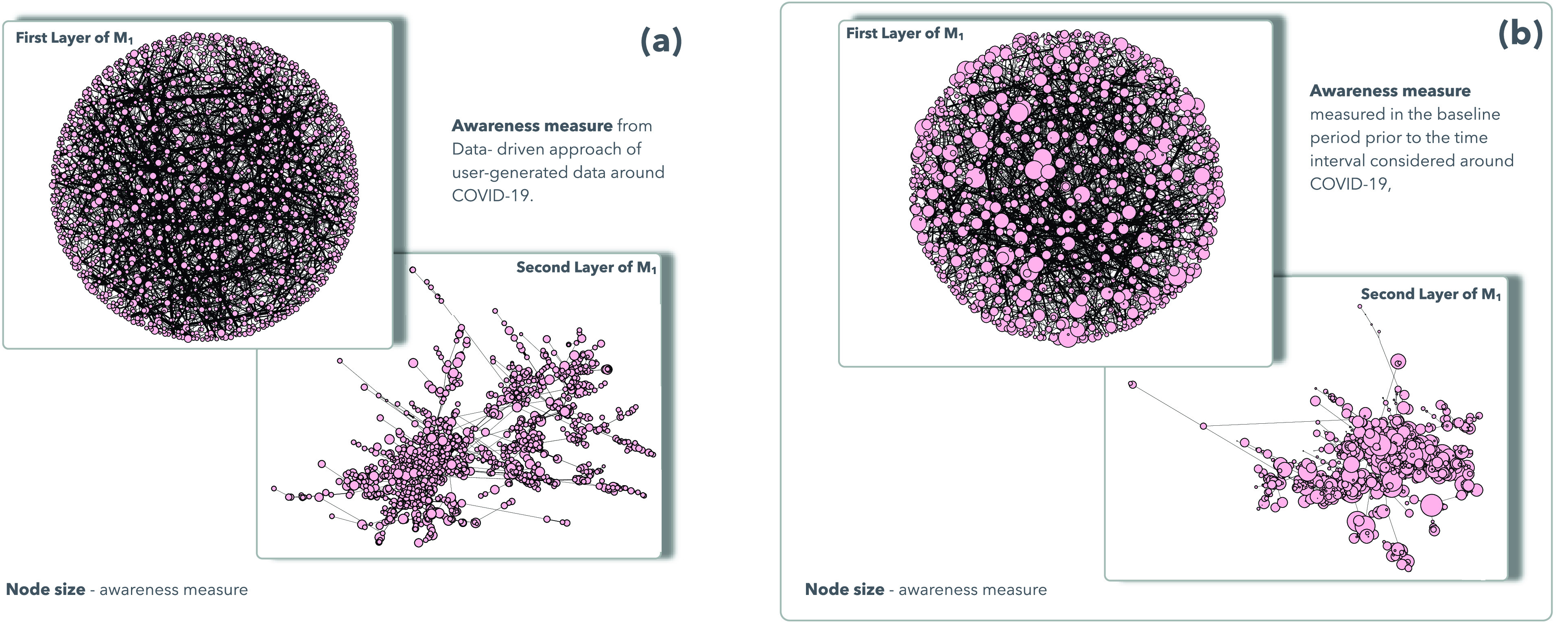
Awareness distribution on Multiplex Network. We show the heterogeneous distribution of the awareness on the two layers in the weighted multiplex network $M_{1}$. In (a) and (b) we illustrate the two layers of the weighted multiplex network $M_{1}$ (as explained in section III-B1). In the top-left and bottom-left panels, in (a), we respectively show the heterogeneous distribution of the awareness measures, in the first and in the second layer, extracted from the data-driven approach of user-generated data around COVID-19. In the top-right and bottom-right panels, in (b), we respectively shed light on the heterogeneous distribution of the awareness measures, in the the first and in the second layer, in the baseline case. See section IV-A for details about baseline and data-driven comparison method.

From Fig. 3, we can observe how the structural heterogeneity of interactions and the heterogeneous distribution of the awareness characterize our model, depending on the parameters of the multiplex network, considering the features of each node regarding the two co-evolving spreading processes around epidemics and awareness dynamics. Each node belongs to the sampled users set extracted from the data-driven approach, and it owns a distinct awareness measure, such a consciousness that allows to react differently to the epidemics, producing a different susceptibility. Following the assumption of our proposed model and the data mined, in panel (a) of Fig. 3 we highlight the heterogeneity distribution in network structures for the case of awareness measures based on multiplex parameters and user-generated data mined from Twitter and Google Trends (see details in both Sections III-B3 and IV-A), in comparison with the awareness measures based on baseline statistics, as showed in panel (b). It is notable how the multiplex network exhibits a heterogeneous distribution of awareness measures in panel (b), as a result of a global attention distributed in various clusters of different topics. Instead, consequently to the occurrence of an emergency event, as COVID-19, it acts as a shaking force that polarizes the collective attention shaping the awareness distribution in multiplex network $M_{1}$ into a more homogenized structure since the nature of the event rules the choice on what to pay attention [35]. This effect produces a decreasing in randomness and an increasing of a collective process. In accordance with the assumption of our model, the awareness measures distribution acts on the interactions weights in conjunction with the homophily measure, impacting on the heterogeneity in terms of node susceptibility against the epidemics, and in terms of infection and awareness rate, shaping also the structural heterogeneity of the degree distribution in the $M_{1}$ weighted multiplex network.

**FIGURE 4.**
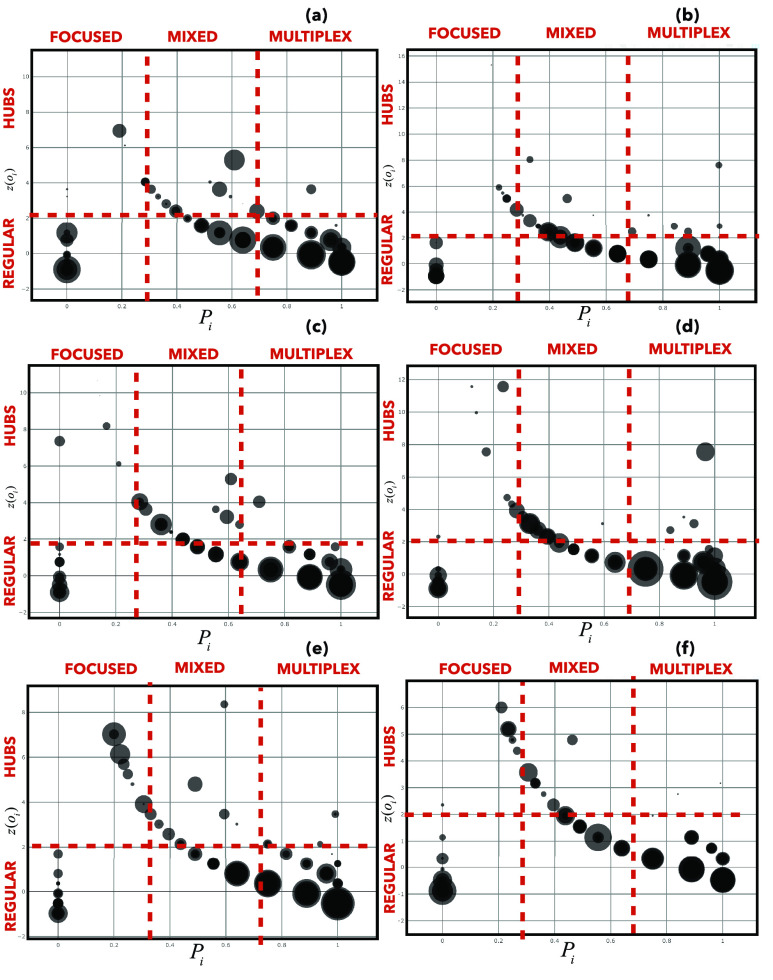
Roles of nodes. We show a cartography of the roles of the nodes in the multiplex network $M_{1}$ obtained by plotting, for each node, the multiplex participation coefficient $P_{i}$ versus the $Z$-score of the total overlapping degree $o_{i}$. Even if two nodes have exactly the same value of $z(oi)$, they can have different roles, as indicated in figure, according to the value of the multiplex participation coefficient. We compare the plots (a)-(c)-(e), which are referred to the case of data-driven awareness measures, with (b)-(d)-(f), which represent the roles of nodes in the baseline case as explained in Section IV-A. We can identify, as put in evidence in the plots, the focused, mixed, multiplex, regular or hubs nodes. The size of each node indicates the awareness measure $aw_{i}$ and its distribution, depending on the collective attention, structurally and dynamically analyzed in $M_{2}$. In both cases, in (a)-(c)-(e) and in (b)-(d)-(f), from top to bottom panels, we decrease the homophily measures among nodes.

In each plot of Fig. 4, the curves correspond to the different values of the distribution of $P_{i}$, the participation coefficient of node $i$ in the weighted multiplex network $M_{1}$ in the range [0, 1], that gives information about the distribution of the edges across the layers for a node $i$. Firstly, we can observe that there is a quite broad distribution suggesting the presence of various levels of node’s participation to each of the two layers in $M_{1}$. We consider this variation in function of the Z-score of the overlapping degree of the node, representing its overall importance in terms of number of edges, with the aim at introducing a classification of nodes in terms of its properties into the multiplex network, highlighting also the awareness distribution, showed as size of nodes. Moreover, the panels (a)-(c)-(e) are referred to the data-driven awareness measures, the panels (b)-(d)-(f) to the baseline statistics, and in both cases from the top to bottom panels we decrease the homophily measures among nodes [31], [78]. We identify in each plot different categories of nodes, hubs, for which $o_{i}\ge 2$, regular nodes, for which $o_{i} < 2$. Representing each node as a point in the plane $P_{i}-Z_{o_{i}}$, by considering the multiplex participation coefficient and the variation in function of $Z_{o_{i}}$, we pick out the six classes of nodes as highlight in Fig. 4, varying homophily measures, showing the awareness distribution. While we observe the same trend in both cases, it is observable the greatest randomness effect, in terms of awareness distribution, in plots referred to the baseline statistics, (b)-(d)-(f). In the case of (a)-(c)-(e) plots, the more participatory the nodes are, the more they acquire a greatest value of awareness, especially in the case of high homophily among nodes (as showed in plot (a)), disclosing this trend especially for regular mixed and multiplex aware nodes. Decreasing homophily values, from (c)-(d) to (e)-(f), respectively referred to awareness data-driven and that one based on baseline statistics, we highlight, with an increasing of $P_{i}$ values, a heterogeneous distribution of awareness in the plane $P_{i}-Z_{o_{i}}$, and a higher density of hubs nodes, in focused roles with higher awareness values. This trend is more evident in the case of awareness based on data and it means that, decreasing the homophily, it is possible to find out a higher presence of focused hubs, more aware in the case of awareness based on data than in baseline statistics case, since the latter suffers the effect of attention randomness. As a result of a collective process around an extraordinary event, in (e) plot we depict this effect, showing how the most aware nodes are either more participatory or more central.

**FIGURE 5.**
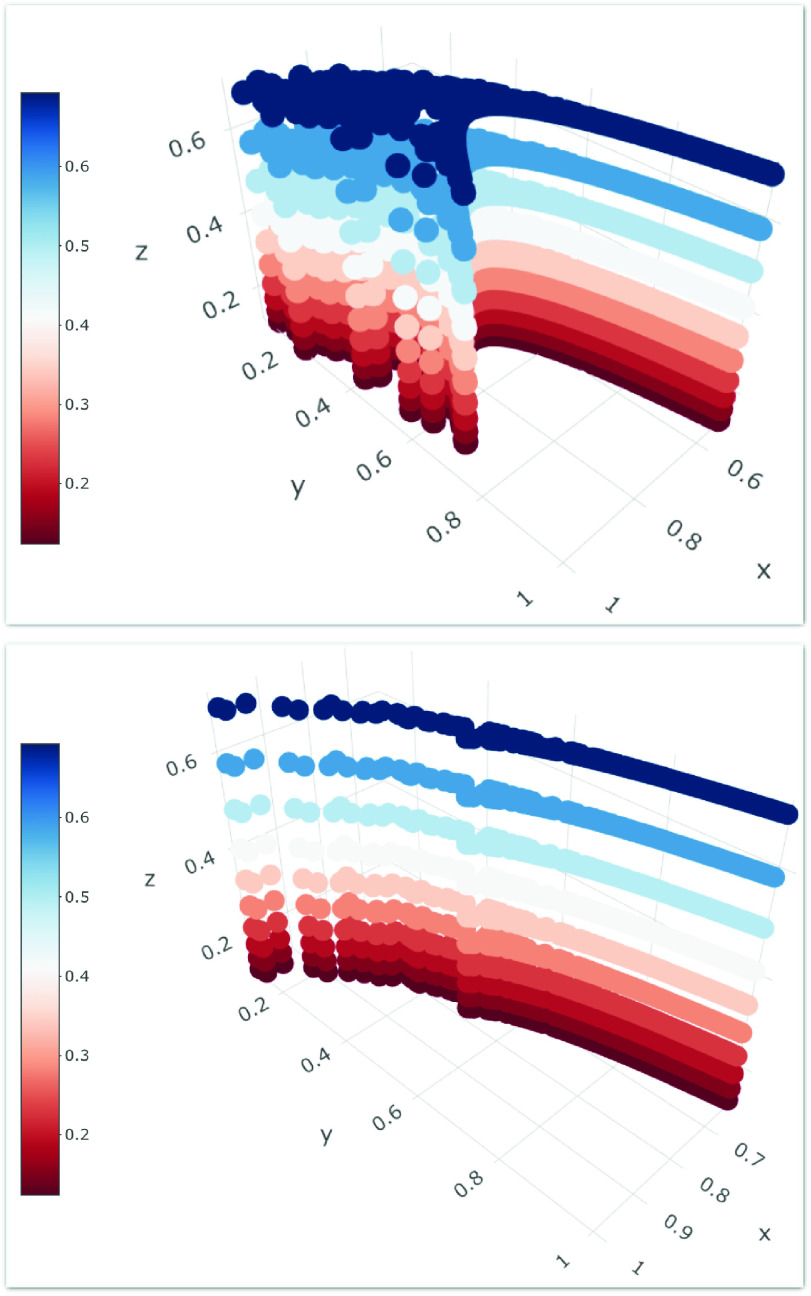
Phase Diagrams $\lambda -\beta -\rho $. Plots, both illustrate in the panel (a) and (b), show the density of infected nodes $\rho $, ranging from red to blue, in function of awareness rate$\lambda $, and the investment fection rate $\beta $, as a result of the dynamical co-evolution of the two spreading processes, epidemics and awareness, in the multiplex network $M_{1}$. We compare the cases of awareness measures $aw_{i}$, extracted from a null model or baseline, as in (a) plot, and the extracted from data-driven approach ones, as showed in (b) plot. Plots are obtained using MMCA method and MC simulations as detailed in Section III-C3.

**FIGURE 6.**
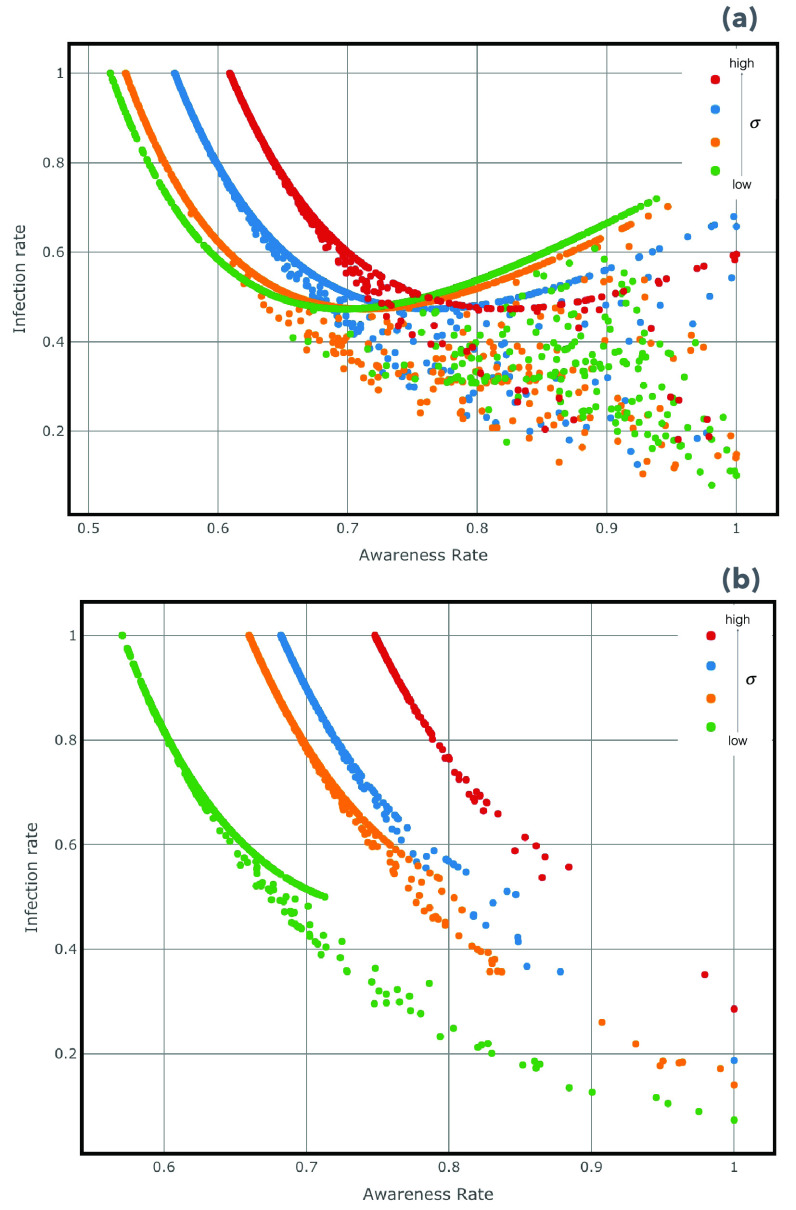
Diagrams $\lambda -\beta $. The plots show, in the plane $\lambda -\beta $, the trend of infection rate in function of awareness rate, respectively in both cases of awareness measures of nodes (a), the baseline case and (b), the data-driven. In both cases we set the following parameters $\beta =0.8$,$\lambda =0.4$, $\delta =0.2$,$\gamma =0.5$, and the standard deviation of homophily value is ranging from a low ($\sigma =0.2$) to high ($\sigma =30$) values (curves from green to red) in both (a) and (b) plots.

In Fig. 5 we display the phase diagram in the plane $\rho -\beta -\lambda $, according to the co-evolving processes of epidemics and awareness in the weighted multiplex network $M_{1}$, and its interdependence with the collective attention dynamics in $M_{2}$. In both panels we show how the double heterogeneity, in terms of infection and awareness rates, allows delaying the contagion outbreak and reducing the density of infected nodes. An increasing of awareness rate results in a decreasing of infection rate up to a specific value of awareness rate, as showed in (a) plot, in which the awareness measures for each node derived from the baseline statistics in a scale-free structure. This means that the remarkable shape is due to the dependence of the heterogeneous susceptibility on the awareness and, when the probability of acquiring more awareness exceed a threshold, this leads to a more probable shift in attention in correlated topics, resulting in an increasing of $\rho $, the density of infection. Differently, in the (b) plot of Fig. 5, which is referred to the case of awareness measures derived from data, that effect vanishes due to more homogenized trend of awareness values around topic linked to the extraordinary event occurrence, and with an increasing of awareness rate we have a decreasing of the epidemics trend. In Fig. 6 we show the trend of infection rate in function of awareness rate, varying the homophily value. The homophily, representing a human-related factor of our modeling approach enables the structural investigation of how the connections are forged in social networks. For that reason, we vary its standard deviation associated to traits, that define the similarity between a pair of nodes showing the resulting trend in the plane $\lambda -\beta $. We take into consideration the two cases of the baseline and the data-driven in reference to the awareness of the users, varying the homophily standard deviation. In the plot (a), referred to the baseline case, we find out that by decreasing the homophily (from green curve to red curve), up to a threshold, the increasing of awareness rate have a minor impact on the infection rate since the population interests are distributed around different topics and the collective attention is shaped as a more heterogenous structure. Differently, in (b), that is based on data mined from Twitter communications under COVID-19, we figure out that by increasing the awareness rate, we can observe a decreasing in infection rate. By decreasing the homophily, the awareness impact is lower than the baseline case, unveiling that the collective dynamics of attention and awareness is more homogenous around the COVID-19.

**FIGURE 7.**
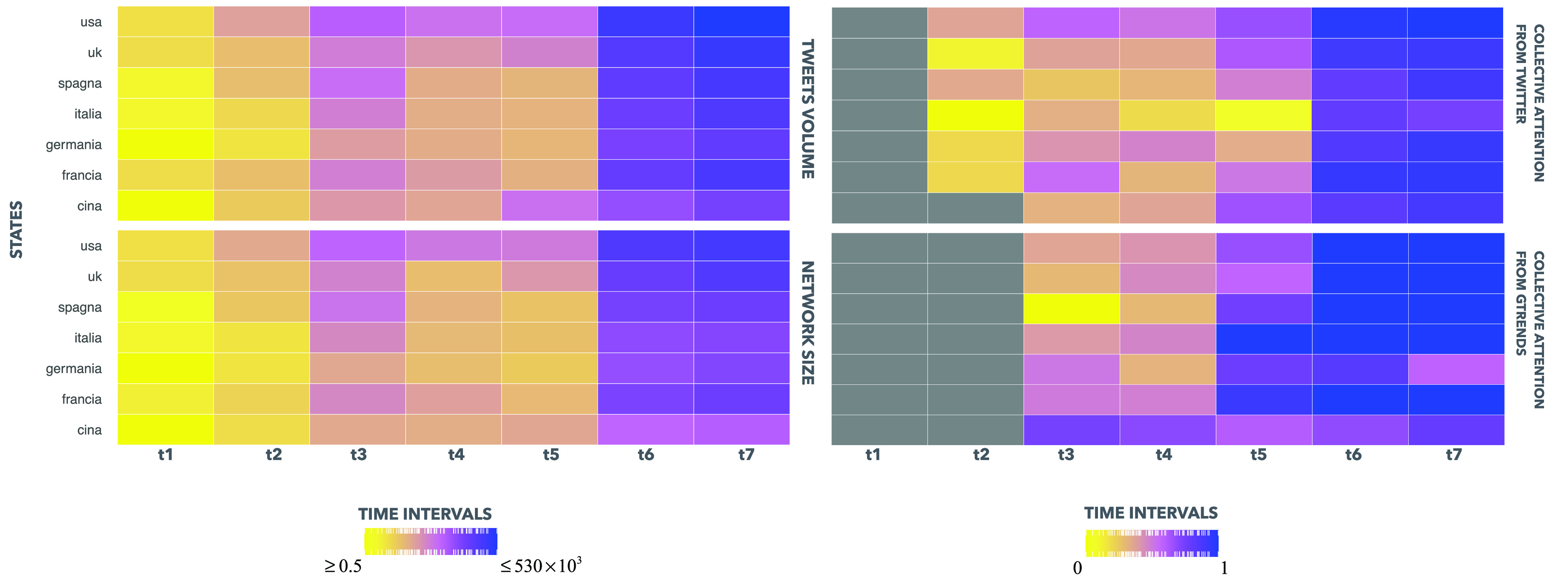
The changes of collective attention from user-generated data during time intervals around COVID-19. For each considered state, on time intervals basis, from $t_{1}$ to $t_{7}$, as argued in section IV-A, we display the heatmap, with colours ranging from yellow to blue, and in accordance with the two referred deviation bars of network metrics values considered in the data-driven approach, as tweets volume, network size, collective attention from Twitter and collective attention from Google Trends. The colour shades of the tweets volume and network size are referred to the entire population observed, differently from the other two metrics, collective attention from Twitter and Google Trends in which we consider the sampled users population. See section IV-A for details.

**FIGURE 8.**
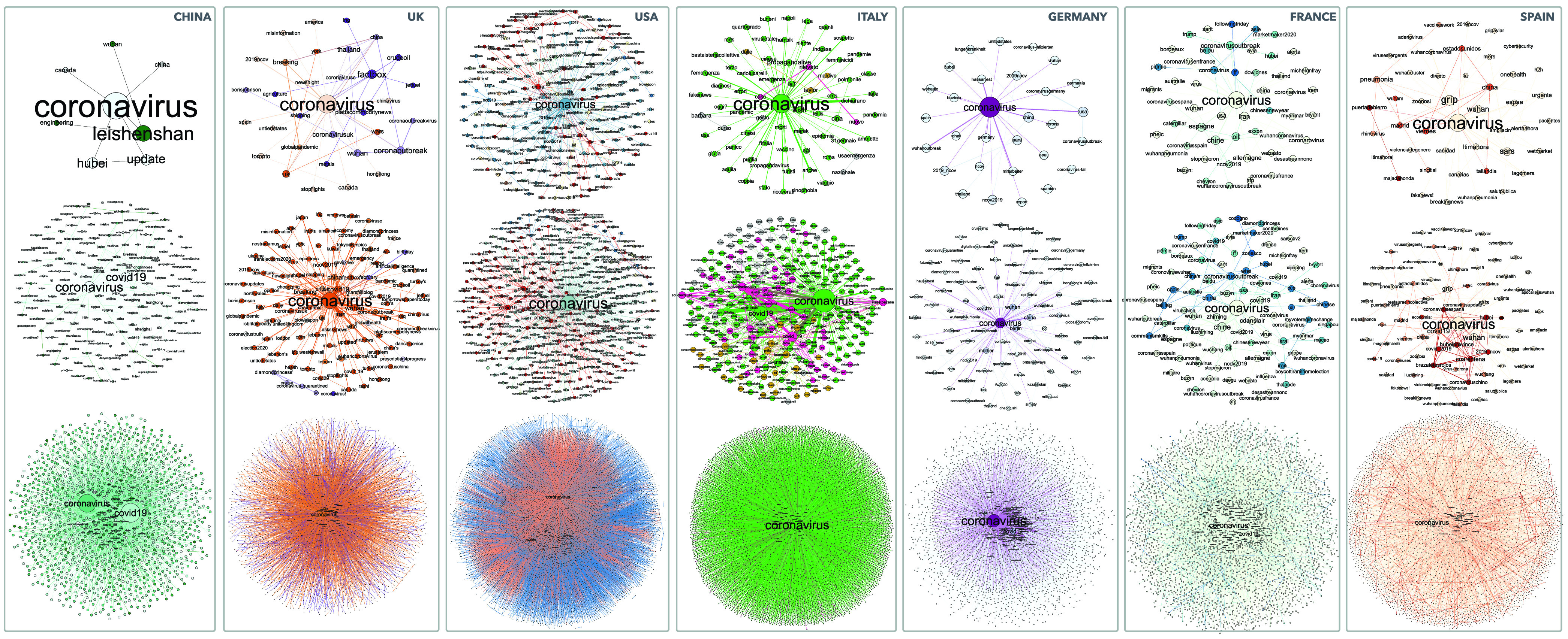
Collective attention and Hashtags co-adoption shifting in time intervals around COVID-19. We display three temporal changes, referred to time intervals $t_{3}-t_{5}-t_{7}$, respectively corresponding to graphs in top, middle, bottom, for each state, of the weighted multiplex $M_{2}$, composed by seven layers, represented by the co-adoption network graph, extracted from Twitter user-generated data, from sampled users come from each state considered in the model (CHINA, UK, USA, ITALY, GERMANY, FRANCE, SPAIN).

**FIGURE 9.**
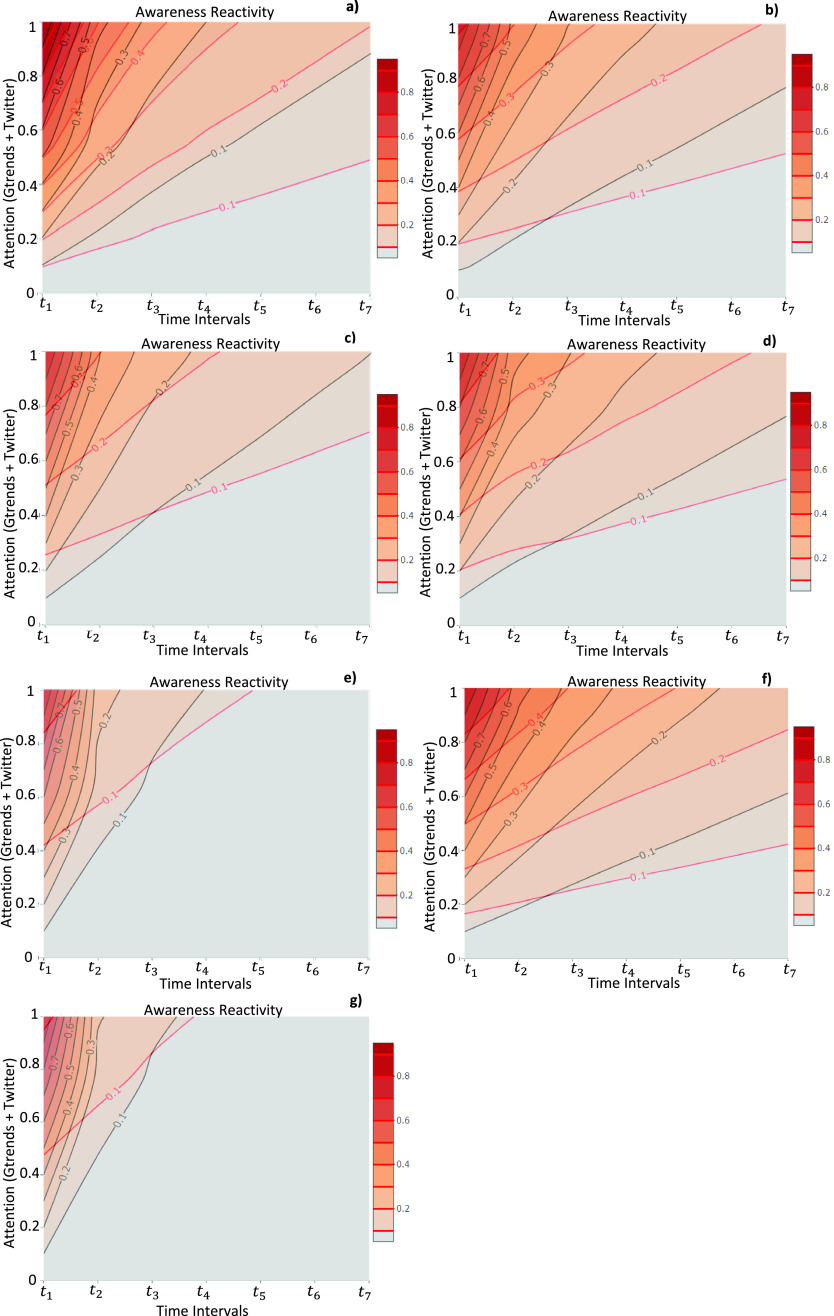
Social marker impact on the Awareness Reactivity Growth Rate. The contour plots show the awareness reactivity changes and the social marker impact on the different time windows in function of the collective attention extracted through the data retrieved from Twitter and Google Trends. Each plot is referred to the different states under investigation ((a)China, b) France, c) UK, d) Germany, e) Spain, f) USA, g) Italy), the red lines represents the values of awareness reactivity and the black lines the values of awareness reactivity taking into consideration in its computation the impact of the social marker. The area enclosed between them is its variation, in terms of growth rate or decrease.

In Fig. 7, we exploit an heatmap to summarize the collective attention dynamics, during temporal window $T$, divided in seven-time intervals, as detailed in Table 4, around COVID-19. The heatmap is based on user-generated data from Twitter communications and queries from Google Trends (see Section IV-A). This plot allows for comparing different network metrics, such as tweets volume, networks size, collective attention from Twitter and Google Trends, ranging the different normalized values into coloured bands. We depict the different colour shades in the bands for each geographical state considered, in reference to each time interval to underline the changes in time, in correlation with the red points, that indicates in which time interval falls the first case of COVID-19 officially, reported to WHO. The grey bands point out the lack of values on collective attention, in the range that covers $t_{1}$ and $t_{2}$. Following the assumption of our model, this puts in evidence that in the sampled users set we take into consideration users which have a role in the rising of collective attention and consequently in the awareness and epidemics dynamics. For that reason, although there are collective attention data on COVID-19 in $t_{1}$ and $t_{2}$, these are not referred to relevant nodes in dissemination. Moreover, we highlight, through the red points, the time in which each state goes from being observer to affected community. Thus, considering the tweets volume and the network size metrics, we shed light on a shifting in trend after $t_{6}$, detecting a delay of two time intervals from the time when the majority of events occurred. Focusing on the collective attention metrics, stated for sampled set of users, we deep inside the patterns of collective dynamics zooming the shape of reaction in response to the events occurred. This shows a growing interest, that although it is not free from delay, follows the timing of COVID-19 appearance. In Fig. 8 we illustrate for each state the evolution of the collective attention, mined from the analysis of user-generated data from Twitter communications, in different crucial time intervals, $t_{3}$, $t_{5}$ and $t_{7}$, that fall within the temporal window $T$. We exhibit the representation of the weighted graph networks captured by the use of different co-adopted hashtags, filtered following the data-driven approach as underlined in Section IV-A. The Fig. 8 shows, looked at it from top to down, the dynamical evolution of co-adoption around the topic, reflecting the scattered attention among various co-adopted hashtags linked to COVID-19 (see Supplementary Information). It highlights a quantitative measure of users’ attended topics, capturing it close to the events in $t_{3}$, after the events happened in $t_{5}$, and finally, when the collective attention get up to speed up to $t_{7}$. The graph networks populate the layers of weighted multiplex networks $M_{2}$, following the assumption of our model (see Section III-B2), introducing quantitative dynamical statistical parameters referred to the collective attention impacting on the dynamical behaviours of the users in the weighted multiplex network $M_{1}$. The Fig. 8 exhibits the rising of the digital traces allowing for exploring the users’ interest into co-adopted various hashtags about COVID-19, representing the willingness to expose their attention to as many people as possible, thus, increasing the likelihood to give a boost to a collective process. The Table 5 lists various information about the response time, in terms of both strategical measures and social collective attention, the occurrence of COVID-19 cases and the awareness reactivity to the emergency occurrence, also with the impact of a social network marker. For the purpose of comparing the public response to the topic around COVID-19 across countries selected, we compare the time of the cases reported to WHO for each country and the peak response in terms of attention, from Twitter and Google Trends, as a result of the analysis of user-generated data. We also consider the “Delay from China reported first case”, which represents the delay of the first response of the collective attention from the China reported first case, the “Starting lockdown-quarantine measures”, which is the time when the countries decide to start the strategical measures, and the “Delay of lockdown measures from the reported internal cases for each country”. Moreover, we define the awareness reactivity $awr_{i}$, for a country $i$ as a statistical parameter which quantify a measure of responsiveness based on information entropy $H$, computed on the basis of data extracted from social media platforms considered, as Twitter and Google Trends, over the elapsed time from first reported case of each country. This parameter weighs the speed at which a country knowingly took notice of the emergency, deciding to start strategical measures, to assess the optimal policies while minimizing the output costs of the protective strategies. High values of awareness reactivity $awr_{i}$ means that there was a fast response to the emergency, that matches high entropy of content shared in online social media platforms and collective attention dynamics, and a high alertness impacting the timing of strategical safety measures. Moreover, taking into consideration the elapsed time between the first peak of the collective attention from the first reported case for each country, we can calculate the awareness reactivity by subtracting this delay from the other delays considered. As indicated in the last column, the resulting value is expressed as an awareness reactivity growth rate, and we find out in most of the cases a percentage of increase of awareness reactivity. In case of China, we see a percentage in decrease of awareness reactivity since it is the country that has the first peak of collective attention response after its first case reported in WHO, differently from the other countries selected. This value represent the impact on the awareness reactivity, finding out the speeding up of the preparedness and responsiveness that would have had if it had considered the effects of the collective attention and awareness dynamics, that is a rising social measure of the public interest around an emergency that would soon have arrived. The figure 9 shows the $awr_{i}$ changes and the social marker impact on its growth percentage on the different time windows considered (see Table 4) in function of the collective attention extracted from the data-driven approach. The figure graphically displays the variation of the $awr_{i}$ with regards to COVID-19 based on awareness dynamics, considering the impact of the social marker (black line) and not (red line). In particular, the figure represents a prediction of how the $awr_{i}$ for each state would increase or decrease, graphically representing the impact of the social marker, if the attention peak would have been in different time intervals. As we can see, in the cases b), c), d), e), f), g) in the time intervals previous respect to the first reported case, when the states are in the condition of observer the social marker’s effect produces an increase in $awr_{i}$ and when the states become affected causes a decrease over time. The only exception is represented by China (a) which is affected in the whole-time interval under investigation and has a peak of attention that is always legging behind the first reported case to the WHO. In this case, taking into consideration the effect of the social marker, the $awr_{i}$ decreases from the first time interval.

**TABLE 5:** Response Time and Awareness Reaction Time

States	China Reported to WHO	Internal cases reported to WHO	First Peak Response (from Twitter)	First Peak Response (from Google Trends)	Delay from China Reported First Case	Start Lockdown - Quarantine Measures	Delay of lockdown/quarantine measures from first internal case	Awareness Reactivity	Awareness Reactivity(+/−) social marker impact (growth rate in %)
China	31-Dec-2019	–	13-Jan-2020	17-Jan-2020	13 days	23-Jan-2020	23 days	0.93	−26%
USA	–	23-Jan-2020	04-Jan-2020	19-Jan-2020	4 days	(19–24)-Mar-2020	(56–61) days	(0.70 - 0.65)	+46% - +41%
UK	–	01-Feb-2020	13-Jan-2020	20-Jan-2020	13 days	23-Mar-2020	51 days	0.73	+39%
Italy	–	31-Jan-2020	13-Jan-2020	21-Jan-2020	13 days	9-Mar-2020	38 days	0.57	+49%
Germany	–	28-Jan-2020	13-Jan-2020	19-Jan-2020	13 days	23-Mar-2020	55 days	0.45	+28%
France	–	25-Jan-2020	13-Jan-2020	20-Jan-2020	13 days	17-Mar-2020	52 days	1	+22%
Spain	–	01-Feb-2020	01-Jan-2020	20-Jan-2020	01 days	14-Mar-2020	42 days	0.86	+258%

## V. Conclusion

Our work has investigated and modelled a systematic methodology, resulting from a complex modeling approach. Exploiting the social networks and communications technologies, we have proposed the understanding of interplay between the collective attention dynamics and the two co-evolving spreading processes as awareness and epidemics, in two interdependent and heterogeneous weighted multiplex networks, under emergency situations in consequence of the occurrence of an extraordinary event as the ongoing COVID-19 epidemics. The findings demonstrate how the proposed modeling and data-driven approaches, and the mathematical framework represent a complex digital observatory to detect a social network marker exploting the digital traces of behaviours and connections of people, depicting the possible collective social response. We identify from dynamical collective patterns, social networks markers to optimally schedule effective crisis communications, facilitating timely crisis response planning, such as the decision of a time warning, based on the trends of attention and awareness. The rising of the collective attention in case of shocking event, as a terrorist attacks, mass shootings or earthquakes takes place immediately during or at the end of the event, and the fast awareness acquired on the circumstances could have the strength to limit the consequences, but does not change the event. Differently, a case similar to a long-lasting event as COVID-19 epidemics, the acquisition of awareness over time, conveyed by the attention and its maintenance, could change the flow of epidemics. This can improve the future preparedness plans, risk factors assessment and actionable strategies for delaying or stopping the spreading. In our work, the non-physical distancing factors, as attention and awareness play an important part in mitigating the spreading, and especially when physical distancing measures are relaxed, the dynamics of collective behaviours in social networks can produce a flow of information that can lead to a shape of attention to be allocated in an efficient way for the affected communities. Physical social distancing strategies, although we do not assume in our work, represent a key point for our future research that could be directed towards the understanding of the role of the location-specific physical distancing policies and the joint impact of real and virtual collective dynamics on awareness. As future work, we plan to conduct a more comprehensive study on collective attention network, comparing different types of events, and the co-occurrence of some of them. This offers more insights on human behaviours and interests leading to an optimally management of the dissemination and the discrimination of the contents, by including mechanisms to improve users’ cognitive ability. Moreover, weighing the dynamics of real and virtual conditions, considering physical strategies, as the social distancing, and non-physical strategies, we will aim at detecting the configurations of social dilemma and network structures that conduct to the emergence and sustainability of human cooperation. This can result in an increasing of discriminating capacity for users to identify better information in comparison with low-quality ones or misinformation. We envision to consider clustering methods and community detection algorithms, in order to introduce cluster-specific preparedness plans.

## Code Availability

To build the model, do computation and obtain our results we used the programming language R and the IDE RStudio. The figures were generated thanks to the package Plotly and the software Gephi [79]–[82]. The developed methodology in terms of multiplex representation, spreading processing modeling and data-driven approach applied to the user-generated data extracted from Twitter communications and Google Trends, under COVID-19 epidemics, is available to the scientific community at the following repository: https://github.com/UnictScata/Collective-Attention-Awareness-and-Epidemics-Spreading-in-the-Multiplex-Social-Network.
